# Unveiling a novel cancer hallmark by evaluation of neural infiltration in cancer

**DOI:** 10.1093/bib/bbaf082

**Published:** 2025-03-07

**Authors:** Qi Dong, Yingying Guo, Chen Lv, Lingxue Ren, Bo Chen, Yan Wang, Yang Liu, Mingyue Liu, Kaidong Liu, Nan Zhang, Linzhu Wang, Shaocong Sang, Xin Li, Yang Hui, Haihai Liang, Yunyan Gu

**Affiliations:** Department of Systems Biology, College of Bioinformatics Science and Technology, Harbin Medical University, No. 157 Baojian Road, Nangang District, Harbin 150081, China; Department of Biochemistry and Molecular Biology, College of Basic Medical Sciences, Harbin Medical University, No. 157 Baojian Road, Nangang District, Harbin 150081, China; Department of Systems Biology, College of Bioinformatics Science and Technology, Harbin Medical University, No. 157 Baojian Road, Nangang District, Harbin 150081, China; State Key Laboratory of Frigid Zone Cardiovascular Diseases (SKLFZCD), Department of Pharmacology (State-Province Key Laboratories of Biomedicine-Pharmaceutics of China, Key Laboratory of Cardiovascular Research, Ministry of Education), College of Pharmacy, Harbin Medical University, No. 157 Baojian Road, Nangang District, Harbin 150081, China; Department of Systems Biology, College of Bioinformatics Science and Technology, Harbin Medical University, No. 157 Baojian Road, Nangang District, Harbin 150081, China; State Key Laboratory of Frigid Zone Cardiovascular Diseases (SKLFZCD), Department of Pharmacology (State-Province Key Laboratories of Biomedicine-Pharmaceutics of China, Key Laboratory of Cardiovascular Research, Ministry of Education), College of Pharmacy, Harbin Medical University, No. 157 Baojian Road, Nangang District, Harbin 150081, China; Department of Systems Biology, College of Bioinformatics Science and Technology, Harbin Medical University, No. 157 Baojian Road, Nangang District, Harbin 150081, China; State Key Laboratory of Frigid Zone Cardiovascular Diseases (SKLFZCD), Department of Pharmacology (State-Province Key Laboratories of Biomedicine-Pharmaceutics of China, Key Laboratory of Cardiovascular Research, Ministry of Education), College of Pharmacy, Harbin Medical University, No. 157 Baojian Road, Nangang District, Harbin 150081, China; State Key Laboratory of Frigid Zone Cardiovascular Diseases (SKLFZCD), Department of Pharmacology (State-Province Key Laboratories of Biomedicine-Pharmaceutics of China, Key Laboratory of Cardiovascular Research, Ministry of Education), College of Pharmacy, Harbin Medical University, No. 157 Baojian Road, Nangang District, Harbin 150081, China; Department of Systems Biology, College of Bioinformatics Science and Technology, Harbin Medical University, No. 157 Baojian Road, Nangang District, Harbin 150081, China; Department of Systems Biology, College of Bioinformatics Science and Technology, Harbin Medical University, No. 157 Baojian Road, Nangang District, Harbin 150081, China; Department of Systems Biology, College of Bioinformatics Science and Technology, Harbin Medical University, No. 157 Baojian Road, Nangang District, Harbin 150081, China; State Key Laboratory of Frigid Zone Cardiovascular Diseases (SKLFZCD), Department of Pharmacology (State-Province Key Laboratories of Biomedicine-Pharmaceutics of China, Key Laboratory of Cardiovascular Research, Ministry of Education), College of Pharmacy, Harbin Medical University, No. 157 Baojian Road, Nangang District, Harbin 150081, China; Department of Systems Biology, College of Bioinformatics Science and Technology, Harbin Medical University, No. 157 Baojian Road, Nangang District, Harbin 150081, China; Department of Systems Biology, College of Bioinformatics Science and Technology, Harbin Medical University, No. 157 Baojian Road, Nangang District, Harbin 150081, China; Department of Biochemistry and Molecular Biology, College of Basic Medical Sciences, Harbin Medical University, No. 157 Baojian Road, Nangang District, Harbin 150081, China; State Key Laboratory of Frigid Zone Cardiovascular Diseases (SKLFZCD), Department of Pharmacology (State-Province Key Laboratories of Biomedicine-Pharmaceutics of China, Key Laboratory of Cardiovascular Research, Ministry of Education), College of Pharmacy, Harbin Medical University, No. 157 Baojian Road, Nangang District, Harbin 150081, China; Department of Nephrology, Second Affiliated Hospital of Harbin Medical University, No. 246 Xuefu Road, Nangang District, Harbin 150081, China; Department of Systems Biology, College of Bioinformatics Science and Technology, Harbin Medical University, No. 157 Baojian Road, Nangang District, Harbin 150081, China

**Keywords:** neural infiltration, cancer hallmark, tumor microenvironment, single-cell RNA sequencing, immunotherapy

## Abstract

Cancer cells acquire necessary functional capabilities for malignancy through the influence of the nervous system. We evaluate the extent of neural infiltration within the tumor microenvironment (TME) across multiple cancer types, highlighting its role as a cancer hallmark. We identify cancer-related neural genes using 40 bulk RNA-seq datasets across 10 cancer types, developing a predictive score for cancer-related neural infiltration (C-Neural score). Cancer samples with elevated C-Neural scores exhibit perineural invasion, recurrence, metastasis, higher stage or grade, or poor prognosis. Epithelial cells show the highest C-Neural scores among all cell types in 55 single-cell RNA sequencing datasets. The epithelial cells with high C-Neural scores (epi-highCNs) characterized by increased copy number variation, reduced cell differentiation, higher epithelial–mesenchymal transition scores, and elevated metabolic level. Epi-highCNs frequently communicate with Schwann cells by FN1 signaling pathway. The co-culture experiment indicates that Schwann cells may facilitate cancer progression through upregulation of *VDAC1*. Moreover, C-Neural scores positively correlate with the infiltration of antitumor immune cells, indicating potential response for immunotherapy. Melanoma patients with high C-Neural scores may benefit from trametinib. These analyses illuminate the extent of neural influence within TME, suggesting potential role as a cancer hallmark and offering implications for effective therapeutic strategies against cancer.

## Graphical Abstract

**Graphical Abstract:**
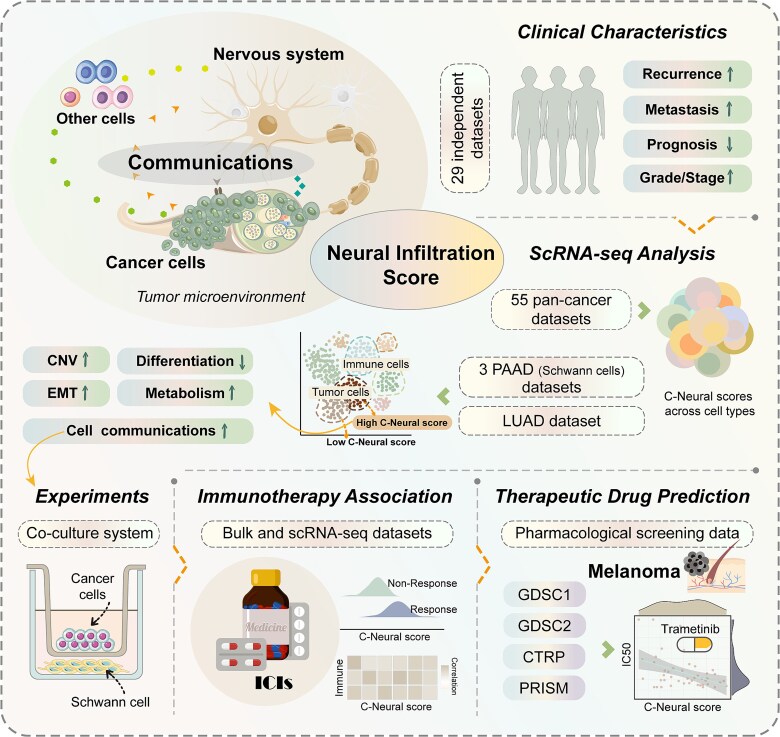

## Introduction

The concept of cancer neuroscience summarizes the reciprocal interactions between the nervous system and cancer [1]. The communication from the nervous system to cancer involves electrochemical signals, paracrine factors, neurotransmitters or neuropeptides. Neuronal activity-dependent paracrine signaling of NLGN3, BDNF, and GRP78 stimulates glioma proliferation and growth [2, 3]. Nerves secrete neurotrophins such as GDNF, ARTN, and NGF that promote nerve–cancer interactions; BDNF/NTRK signaling implicates in promoting tumor survival [4]. Sensory nerves release the neuropeptide substance P to drive breast cancer (BC) growth, invasion, and metastasis [5]. The excitatory neurotransmitter glutamate activates NMDA receptors was evident in the aggressive BC. Besides, targeting glutamatergic neuron-to-glioma synapses by AMPAR inhibitor is an emerging cancer therapeutic strategy [6]. Thus, nervous system and cancer interactions can regulate oncogenesis, growth, invasion and metastatic spread, treatment resistance, stimulation of tumor-promoting inflammation, and impairment of anticancer immunity, which may be a potential, new hallmark of cancer [4]. A thorough investigation and assessment are essential to gain a comprehensive understanding of the effects of neural factors on cancer cells.

Perineural invasion (PNI) was broadly defined as tumor cells appear within any of the three layers of the nerve sheath or as tumor cells surround a nerve closely and involve at least 33% of its circumference [7]. PNI occurs in multiple cancer types associated with tumor progression, recurrence, metastasis, unfavorable outcomes, and reduced survival rates [8]. However, surgical resection may overlook PNI; hematoxylin & eosin (H&E) alone reduced the detection of PNI compared with H&E + immunohistochemistry (IHC) [9, 10]. Additionally, even in PNI-negative oral squamous cell carcinoma, shorter nerve–tumor distance and larger nerve diameter are associated with poor survival, highlighting neural influences on cancer beyond traditional PNI assessments [10]. Peripheral nerves are surrounded by Schwann cells, which can secrete cytokines to promote cancer progression in a variety of cancer types [11, 12]. The current researches exhibit a deficiency in systematic assessments of neural infiltration within the tumor microenvironment (TME), spanning a diverse spectrum of malignancies.

Winkler *et al.* [4] expanded the concept of cancer neuroscience with new multidisciplinary research sub-fields, such as neuro-immuno-oncology. Interactions among nerve cells, cancer cells, and immune cells can modulate the immune microenvironment [13]. For example, peripheral nerve fibers release calcitonin gene-related peptide, which led to a reduction in effector T lymphocytes [14]. Schwann cells promoted M2 polarized macrophages, accelerating lung cancer growth [15]. Neural infiltration in the TME may influence the efficacy of immune checkpoint inhibitors (ICIs). Single-cell RNA sequencing (scRNA-seq) technology enables the detection of cellular heterogeneity and the elucidation of intricate cell–cell interactions. scRNA-seq is instrumental in delineating the neural signals that drive cancer progression.

Transcriptome sequencing technologies facilitate the excavation of biological signature gene sets [16]. The enrichment scores computed by gene set variation analysis (GSVA) based on ranked concerned signature gene sets exhibit greater biological interpretability and robustness among datasets [17]. Wang *et al.* [18] defined cellular senescence score by combining multiple cellular senescence-related genes using enrichment scores. Additionally, the UCell method was designed for scRNA-seq data and computes gene signature enrichment score depending on the relative gene expression in individual cells. Thus, gene set enrichment methods contribute to comprehensive assessments of the neural infiltration in TME.

This study propose a predictive score for cancer-related neural infiltration (C-Neural score) across multiple cancer types. We assessed the association of C-Neural score with TME components and clinical characteristics. Additionally, we explored the malignancy of epithelial cells with discrepant C-Neural scores and their communications with other cells in TME. Finally, we investigated the association of C-Neural score with immunotherapy response and predicted potential drugs for cancer treatment. Our work evaluated neural infiltration levels in cancer, revealing the indicative role of C-Neural score in cancer malignancy and treatment strategies.

## Materials and Methods

### Pan-cancer bulk RNA-seq datasets

Normalized gene expression profiles for 10 cancer types with matching normal samples were obtained from multiple resources (Table S1). A cohort of 40 paired distinct expression profiles encompassing both neoplastic and non-neoplastic samples was assembled.

### Identification of cancer-specific differentially expressed neural genes

The neural genes were primarily collected from four sources (Table S2). After removing duplicates, 1889 neural genes were retained (Table S3). For each paired gene expression profiles, one-sided Wilcoxon rank sum-test was used to identify significantly differentially expressed neural genes (DEG-Ns) (*P* < .01) (Table S4).

### Evaluation of neural infiltration

For bulk RNA-seq data, we computed the C-Neural score using GSVA method with R package “GSVA.”


$$ C- Neural\ score= GSV{A}_{\left( DEG- Nup\right)}- GSV{A}_{\left( DEG- Ndown\right)} $$

For scRNA-seq data, we evaluated the C-Neural score using the UCell algorithm with the R package “UCell.”


$$ C- Neural\ score= UCel{l}_{\left( DEG- Nup\right)}- UCel{l}_{\left( DEG- Ndown\right)} $$

Samples were defined into high C-Neural score group and low C-Neural score group according to the median value.

### Association of C-Neural score with cancer hallmarks and clinical characteristics

Cancer hallmark gene sets were collected from public resources. GSVA method was used to evaluate the cancer hallmark scores for The Cancer Genome Atlas (TCGA) samples. Gene expression data and clinical information for 10 cancer types from 31 datasets were obtained from databases and papers (Table S5). In HNSC_TCGA dataset, we assessed the difference in C-Neural score between PNI-positive and PNI-negative tumor samples.

### Pan-cancer scRNA-seq data processing

A total of 55 scRNA-seq datasets for 10 cancer types were collected (Table S7). The scRNA-seq data from each study was separately processed using R package “Seurat.” R package “harmony” was used to integrate cells across individuals. Cluster-specific genes were identified, and the clusters were annotated by canonical cell markers.

The scRNA-seq data from four pancreatic ductal adenocarcinoma (PDAC) tissues with Schwann cells accumulation was obtained from Xue *et al.* [19]. The scRNA-seq data of 17 pancreatic adenocarcinoma (PAAD) samples and spatial transcriptome data were obtained from Kim *et al.* [20]. The scRNA-seq data were processed using Seurat method. Clusters were annotated by canonical cell markers. Cancer-associated fibroblasts (CAFs) were classified into myofibroblastic CAF, inflammatory CAF (iCAF), and reticular-like CAF (r-lCAF). The epithelial/ductal cell population was divided into high C-Neural score (epi-highCN) and low C-Neural score (epi-lowCN) populations according to the median. In addition, the single-nucleus RNA sequencing data with cell type annotation of 15 PDAC samples was obtained from Single Cell Portal (SCP1089). Tumor cells were categorized into high C-Neural score population (tumor cell-highCNs) and low C-Neural score population (tumor cell-lowCNs) based on the median.

Salcher *et al.* [21] aggregated 29 publicly available datasets to construct a non-small cell lung cancer (NSCLC) single-cell atlas. We downloaded the data with cell type annotation information and extracted cells from 156 lung adenocarcinoma (LUAD) samples. Tumor cells were grouped into tumor cell-highCNs and tumor cell-lowCNs.

### Distribution of C-Neural score in pan-cancer scRNA-seq datasets

For each scRNA-seq dataset, we calculated the C-Neural score for all cells and grouped them based on the median score. For each cell type in a dataset, we computed the distribution proportion in high C-Neural score population (P-high_t_) and low C-Neural score population (P-low_t_). We calculated the fold change (FC) value between P-high_t_ and P-low_t_. For cell types annotated in multiple datasets, we obtained multiple P-high_t_ and P-low_t_ values. One-sided Wilcoxon rank-sum test was executed to assess difference in the distribution of P-high_t_ values and P-low_t_ values.

### Cell subpopulations analysis

To detect malignant cancer cells, we utilized CopyKAT algorithm to estimate single-cell copy number variation (CNV) landscapes by R package “copykat.” Epithelial cells were defined as aneuploid (malignant) and diploid (non-malignant) cells. The CytoTRACE algorithm was employed to estimate the differentiation (stemness) status of cells using R package “CytoTRACE,” with higher CytoTRACE score signifying poor differentiation. R package “slingshot” was used to infer cell lineage and pseudotime. We utilized R package “scMetabolism” to assess the metabolic pathway activity of cells [22]. Epithelial–mesenchymal transition (EMT) genes were sourced from the EMTome database. We evaluated the EMT scores for cells using R package “AUCell” based on the expression of 814 EMT genes.

### Cell–cell communication analysis

Cell–cell communications were inferred using R package “CellChat.” We focused on the human database in CellChat to identify overexpressed ligands or receptors. The cell–cell communications at the signaling pathway level between each cell type was inferred by assigning probability values to each interaction and performing permutation tests. Furthermore, we downloaded protein–protein interactions (PPIs) from the Pathway Commons database. Seurat method was used to identify upregulated genes for epi-highCNs and Schwann cells. The PPIs of upregulated genes were extracted to generate a PPI network. CellChat was then applied to identify significant PPIs.

### Co-culture system and conditioned medium collection

For the co-culture assay, we used a 0.4-μm pore Transwell chamber (Corning, USA). After transfection of siVDAC1/siNC for 24 hours in Panc-1/A549, digested Panc-1/A549 cells (4 × 10^5^) were seeded into the upper Transwell membrane. Then, 4 × 10^5^ sNF96.2 cells were plated in the bottom chamber of 6-well plates. The cells were co-cultured for 48 hours and were used for future study. To collect the Schwann cell-conditioned medium (SC-CM) of sNF96.2 cells, cells were cultured in Dulbecco's Modified Eagle Medium for 48 hours and the supernatant was centrifuged at 2000 × g for 10 minutes to eliminate the cells and cell debris. All the SC-CM were used instantly or frozen at −80°C. For the incubation of Panc-1/A549 cells with SC-CM, Panc-1/A549 cells were incubated with SC-CM for 48 hours. Then, the cells were harvested for future assay.

### Association of C-Neural score with immunotherapy response

Bulk transcriptome data from pre-treatment samples of immunotherapy cohorts were collected (Table S8). Additionally, we obtained scRNA-seq data from three anti-PD-1 pre-treatment NSCLC patients [23]. The Seurat method was used to process scRNA-seq data. The immune checkpoint genes were obtained from Hu *et al*. [24]. The transcriptomic signatures and algorithms for predicting immunotherapy response were collected from published literatures (Table S9).

### Prediction of drug treatment

The pharmacological screening data were downloaded from multiple sources (Table S10). Within each tissue type, we identified pharmacological agents with IC50/AUC/EC50 values exhibiting significant positive or negative correlations with the C-Neural scores. Drug sensitivity values for TCGA samples were inferred using the R package “oncoPredict.” In addition, transcriptome data and drug sensitivity values of 49 melanoma cell lines were obtained from Rydenfelt *et al*. [25]

### Statistics

The one-sided Wilcoxon rank-sum test was applied for comparisons between two groups. The chi-square test was used to assess the categorical difference between groups. Spearman’s rank correlation analysis was utilized to compute the association between two variables. Student’s *t*-test was used to compare the mean values of independent samples. Statistical analyses were conducted using R and GraphPad Prism software. *P* < .05 was considered statistically significant.

### Ethics statement

Human PAAD samples were obtained from the Harbin Medical University Cancer Hospital. All of the patients or their guardians provided written consent. The study was approved by the ethics and scientific committees of the Harbin Medical University Cancer Hospital (KY-2023-41).

See Supplementary Files for further details and abbreviations.

## Results

### C-Neural score indicates cancer malignancy

**Figure 1:**
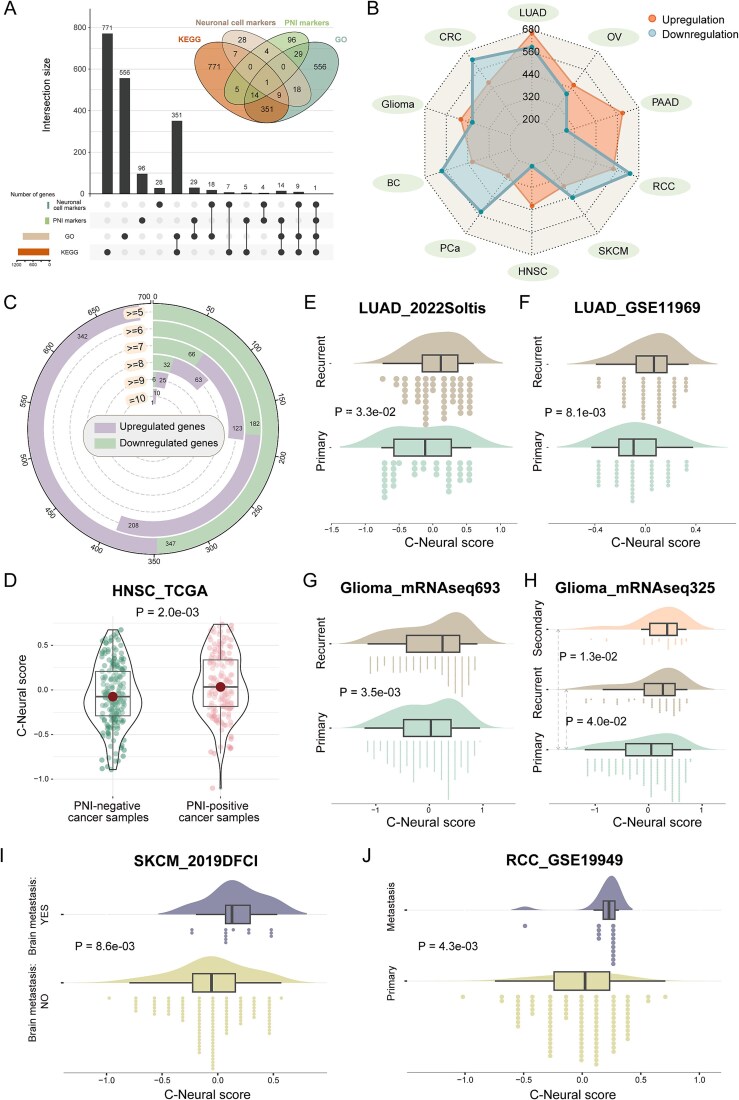
The increased C-Neural scores indicate cancer recurrence and metastasis. (a) Sources of neural genes and the number of overlapping neural genes among sources. (b) Cancer-specific upregulated and downregulated neural genes were identified across 10 cancer types. (c) The overlapping of cancer-specific upregulated and downregulated neural genes among 10 cancer types. (d) Distribution of C-Neural scores between the PNI-positive and PNI-negative HNSC samples. (e)–(g) Distribution of C-Neural scores between primary and recurrent samples. (h) Distribution of C-Neural scores among primary, recurrent, and secondary recurrent cancer samples. (i) Distribution of C-Neural scores between brain metastatic and non-metastatic samples. (j) Distribution of C-Neural scores between primary and metastatic samples. *P*-values were calculated by one-sided Wilcoxon rank-sum test in (d)–(j); *P* < .05 was considered statistically significant.

We evaluated neural infiltration (C-Neural score) in TME across 10 cancer types (Fig. S1A). Neural genes were collected from databases and literature (Fig. 1a). We identified cancer-specific DEG-Ns; some neural genes were consistently differentially expressed across multiple cancer types (*P* < .01; Fig. 1b and c; Fig. S2A). The DEG-Ns that upregulated in at least seven cancer types were significantly enriched in neurodegenerative diseases and cancer hallmarks (Fig. S2B). Neural infiltration levels in cancer samples were computed based on DEG-Ns. The C-Neural scores were positively correlated with cancer hallmark scores in PAAD, kidney cancer, and glioma; hallmarks such as “genome instability and mutation” and “senescent cells” were positively correlated with C-Neural scores in all cancer types (Fig. S2C). In LUAD, BC, and PAAD of TCGA, C-Neural score-related upregulated genes were significantly enriched in cancer hallmark pathways (Fig. S3). We investigated the association between C-Neural scores and clinicopathological features (Fig. S1B). C-Neural scores in PNI-positive head and neck squamous cell carcinoma (HNSC), advanced colon adenocarcinoma, or alive LUAD patients were higher than PNI-negative patients (*P* < .1; Fig. 1d; Fig. S4A and B).

**Figure 2:**
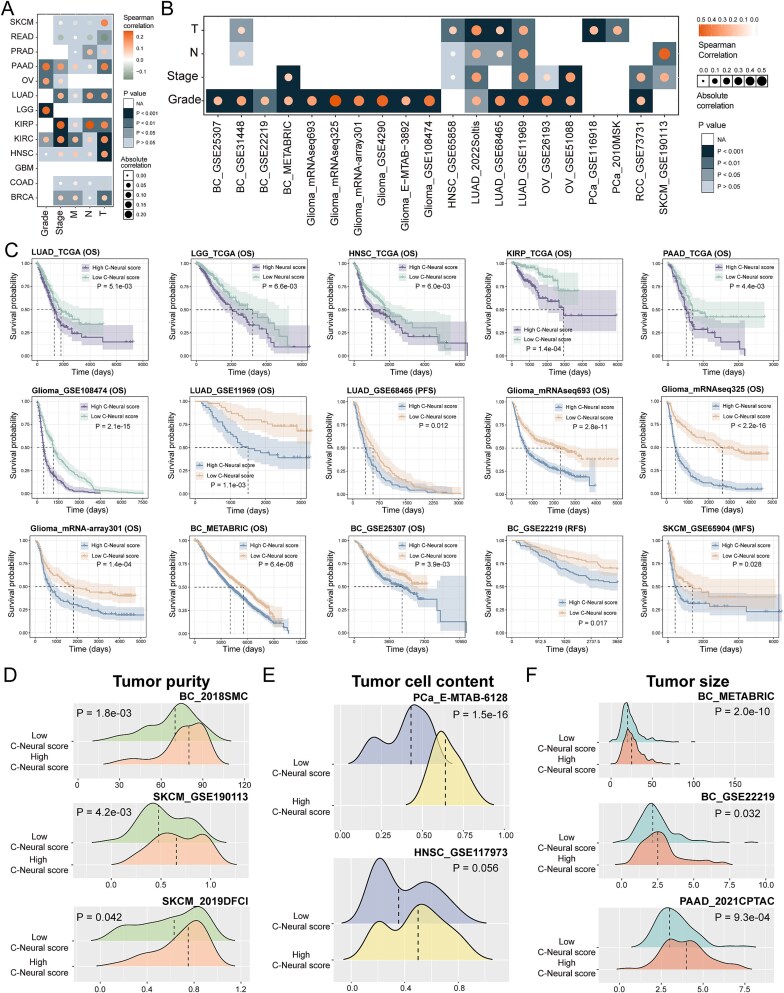
The increased C-Neural scores indicate malignant clinicopathological features, poor prognosis, and highly purity of tumor. (a) Correlation between C-Neural scores and tumor stage or grade in TCGA. (b) Correlation between C-Neural scores and tumor stage or grade in independent datasets. (c) Survival analysis for patients with high and low C-Neural scores in TCGA and independent datasets based on OS, PFS, RFS, and MFS data. (d) Distribution of tumor purity between high and low C-Neural score samples. (e) Distribution of tumor cell content between high and low C-Neural score samples. (f) Distribution of tumor size between high and low C-Neural score samples. *P*-values were calculated by Spearman’s rank correlation analysis in (a) and (b), log-rank test in (c), and one-sided Wilcoxon rank-sum test in (d)–(f); *P* < .05 was considered statistically significant.

For recurrent LUAD and glioma samples, C-Neural scores were significantly higher than that in primary samples (*P* < .05; Fig. 1e–h). Metastatic samples had significantly higher C-Neural scores (*P* < .05; Fig. 1i and j). Moreover, C-Neural scores were positively correlated with stage or grade in most cancer types in TCGA and multiple independent datasets (*P* < .05; Fig. 2a and b; Table S6). Additionally, patients with higher scores had worse prognosis (*P* < .05; Fig. 2c; Fig. S4C).

Then, we explored the level of C-Neural scores in TME components. Samples with high C-Neural scores had elevated tumor purity, tumor cell content, and tumor size (*P* < .05; Fig. 2d–f). In LUAD datasets, C-Neural scores were positively correlated with tumor purity and stemness index and negatively correlated with immune and stromal scores (*P* < .05; Fig. S5A–G). Moreover, C-Neural scores were significantly positively correlated with tumor purity scores that evaluated by IHC and algorithms in eight cancer types of TCGA (*P* < .05; Fig. S5H). Overall, the C-Neural score indicates higher tumor purity and TME components exhibit differential neural signals.

### Pan-cancer scRNA-seq reveals heterogeneity of neural infiltration

**Figure 3:**
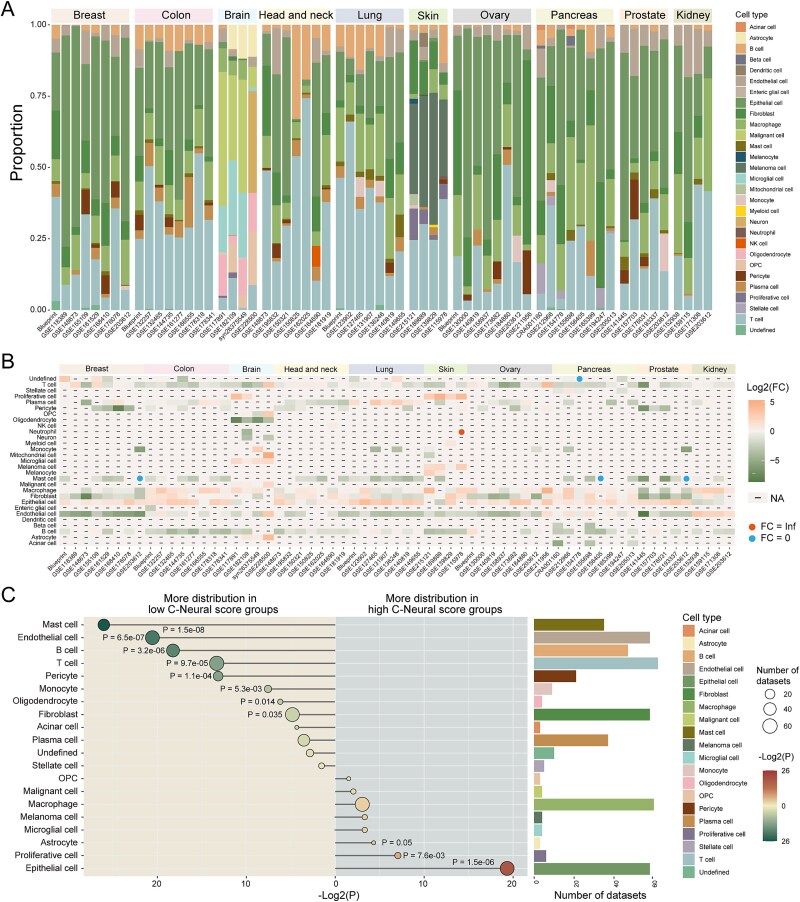
Distribution of C-Neural scores across cell types in pan-cancer scRNA-seq datasets. (a) The bar plot illustrates the proportion of different cell types in pan-cancer scRNA-seq datasets. (b) The heatmap displays the log2(FC) in the number of cell type between high and low C-Neural score cell populations. “-” means the cell type was not identified in the dataset. (c) The difference in cell type proportion between high and low C-Neural score groups in all datasets. The bar plot shows the number of datasets where each cell type was annotated. *P*-values were calculated by one-sided Wilcoxon rank-sum test and *P* < .05 was considered statistically significant.

Given the distinct correlation of TME components with C-Neural score observed in bulk sequencing data, we sought to validate and extend these findings by examining the heterogeneity of C-Neural score levels among different cell types using 55 scRNA-seq datasets (Fig. S1C). After quality control, >80% DEG-Ns were reserved in 96.82% pan-cancer scRNA-seq datasets (Fig. S6A). A total of 28 cell types were annotated across 10 cancer types and assessed the differences in distribution of C-Neural scores (Fig. 3a and b). In most datasets, epithelial cells tend to be distributed in high-score groups, while mast cells, endothelial cells, T cells, and B cells were predominantly found in low-score groups. We revealed significant differences in distributional proportions of cell types between high and low C-Neural score groups across all datasets (*P* < .05; Fig. 3c). Epithelial cells, proliferative cells, and astrocytes showed significantly higher proportions in high C-Neural score groups. Cells such as mast cells and endothelial cells exhibited significantly decreased proportions in high C-Neural score groups. Collectively, the results underscore the diverse neural signals within the TME, with notable enrichment of epithelial cells in high C-Neural score cell population.

### Epi-highCNs display highly malignancy and frequently communicate with Schwann cells

**Figure 4:**
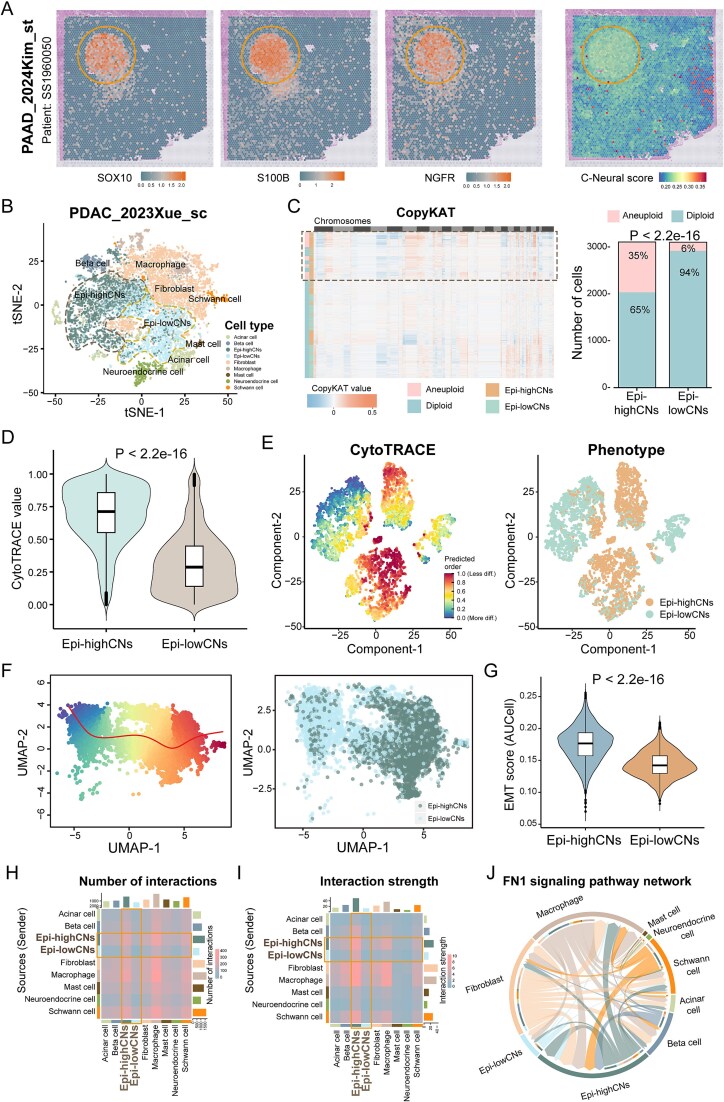
Comparison of epithelial cells with discrepant C-neural scores. (a) The expression of Schwann cell markers (*SOX10*, *S100B*, and *NGFR*) and distribution of C-Neural scores in regions in spatial transcriptome data from a pancreatic cancer sample (SS1960050). (b) Annotation of cell types in PDAC_2023Xue_sc dataset, where epithelial cells were grouped into epithelial/ductal cells and acinar cells. (c) Distribution of inferred CNV between epi-highCNs and epi-lowCNs, with bar colors representing cell groups and differential distribution of aneuploid and diploid cells between epi-highCNs and epi-lowCNs. (d) Distribution of cell differentiation values between epi-highCNs and epi-lowCNs. (e) Cell differentiation levels evaluated by CytoTRACE; the point color means differentiation value. (f) Inferred cell differentiation trajectory by slingshot algorithm and corresponding neural infiltration-related epithelial cell subtypes. (g) Difference in EMT scores between epi-highCNs and epi-lowCNs. (h) and (i) Cell–cell communications among cell types, visualized in heatmaps where color indicates the number or strength of communications. (j) The cell–cell communications of FN1 signaling pathway among cell types. *P*-values were calculated by chi-square test in (c) and one-sided Wilcoxon rank-sum test in (d) and (g); *P* < .05 was considered statistically significant.

PNI is a common characteristic of PAAD and is present in resection region. C-Neural scores were significantly higher in the region that PNI and Schwann cells markers high expressed according to spatial transcriptome RNA sequencing (*P* < 2.2e-16; Fig. 4a; Fig. S6B). We further investigated the discrepancies between epithelial cell subpopulations with distinct neural signals (Fig. S1C). Epithelial/ductal cells were grouped into epi-highCNs and epi-lowCNs subpopulations, which occupied distinct positions in the *t*-distributed stochastic neighbor embedding (t-SNE) plot in the PDAC_2023Xue_sc dataset (Fig. 4b; Fig. S7A–D). Aneuploid cells were significantly enriched in epi-highCNs (*P* < 2.2e-16; Fig. 4c). The CytoTRACE scores of epi-highCNs were significantly greater than epi-lowCNs, with differentiation scores gradually increasing from epi-highCNs to epi-lowCNs (*P* < 2.2e-16; Fig. 4d and e). Combing the inferring pseudotime, we identified a differentiation trajectory from epi-highCNs to epi-lowCNs, suggesting increased stemness in epi-highCNs (Fig. 4f). Additionally, oxidative phosphorylation (OXPHOS) and several metabolic pathways were upregulated in epi-highCNs (Fig. S7E). Epi-highCNs exhibited a significant increase in EMT scores (*P* < 2.2e-16; Fig. 4g; Fig. S8). These analyses highlight the upregulation of cancer hallmarks in epi-highCNs and reveal the potential of C-Neural score to identify malignant epithelial cells.

We explored the discrepancies in cell–cell communications of epi-highCNs and epi-lowCNs in TME. In PAAD scRNA-seq datasets, epi-highCNs exhibited more and stronger communications with other cell types than epi-lowCNs (Fig. 4h and i; Fig. S9), particularly with Schwann cells and neuroendocrine cells (Fig. S10A and B). Epi-highCNs exhibited increased interaction with macrophages and fibroblasts (Fig. 4h and i; Fig. S9). Epi-highCNs, iCAFs, and Schwann cells showed a higher level of interactions in PDAC_2023Xue_sc dataset (Fig. S10C–H). Schwann cells interact with Epi-highCNs by FN1 signaling and collagen-related ligand-receptor pairs (Fig. 4j; Figs S11 and S12). The results revealed that epi-highCNs frequently communicate with other cells, especially fibroblasts. Schwann cells communicated with epi-highCNs by FN1 and collagen signaling in PAAD.

In addition, we identified significant PPIs between Schwann cells and epi-highCNs (Fig. S13A). The proteins in PPI network were significantly enriched in pathways such as “extracellular matrix (ECM)-receptor interaction” and “focal adhesion” (Fig. S13B). Given that interactions between nervous system and cancer cells can promote cancer progression, we hypothesized that patients with co-high expression of notable interactions (*APOD*_*VDAC1*, *APOD*_*PDZK1IP1*, and *CLDN4*_*APOD*) would have poor prognosis. Patients with co-low expression of the gene pairs had significantly improved survival in PAAD datasets (Fig. S13C–G). The results suggest that the PPIs interactions we identified between Schwann cells and epi-highCNs may contribute to cancer progression and poor prognosis.

**Figure 5:**
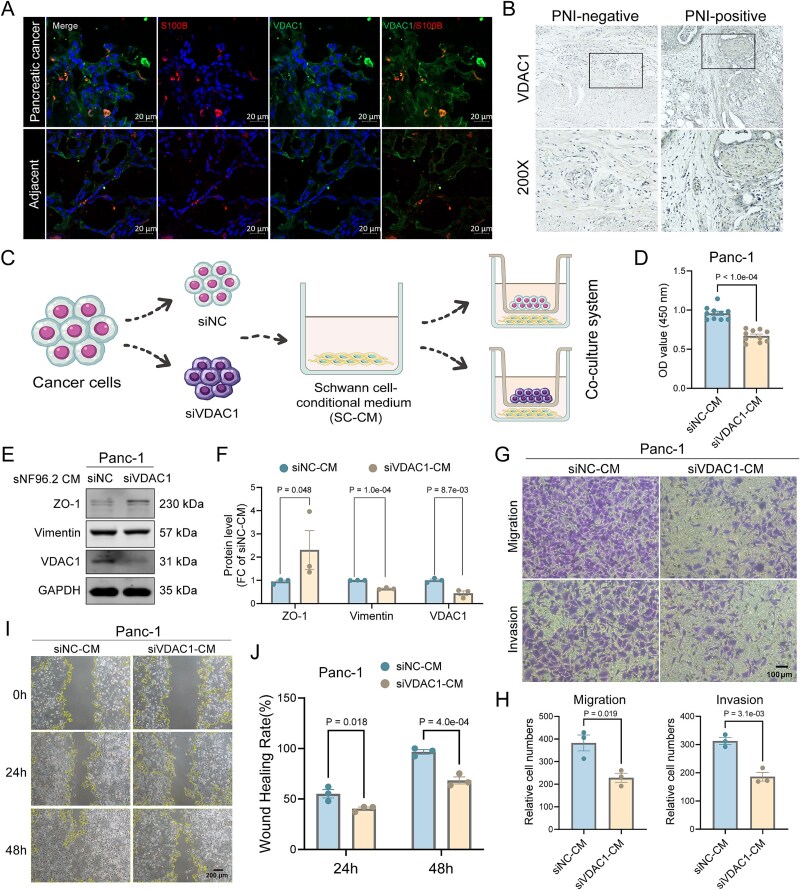
Schwann cells promote PAAD progression mediate by *VDAC1*. (a) Representative immunofluorescence images of Schwann cells with S100B staining and cells with VDAC1 staining in PAAD and adjacent normal tissues. (b) Representative images of IHC staining for VDAC1 in PNI-positive and PNI-negative PAAD tissues. (c) Generation of co-culture systems. (d) CCK8 assay examines the proliferation of Panc-1-siVDAC1 and Panc-1-siNC in SC-CM (*n* = 10). (e) and (f) Western blot analysis for Panc-1-siVDAC1 and Panc-1-siNC in SC-CM (*n* = 3). (g) and (h) Transwell assays for detection of migration and invasion in Panc-1-siVDAC1 and Panc-1-siNC in SC-CM (*n* = 3). (i) and (j) The scratch assays for Panc-1-siVDAC1 and Panc-1-siNC in SC-CM (*n* = 3). *P*-values were calculated by Student’s *t*-test in (d), (f), (h), and (j); *P* < .05 was considered statistically significant.

We investigated the association between Schwann cells and *VDAC1* expression in PAAD tissues. *VDAC1* is primarily expressed in epithelial cells (Fig. S14). In PDAC tissue, VDAC1-expressing cells co-localized with Schwann cells (marked by S100B), whereas were separated with Schwann cells in adjacent normal tissue (Fig. 5a). IHC further confirmed that VDAC1 expression is higher in PNI-positive patients (Fig. 5b). We co-cultured Panc-1 cells that knockdown *VDAC1* (siVDAC1) or siNC with Schwann cells (Fig. 5c). CCK8 assays showed a significant reduction in Panc-1-siVDAC1 cell density in the conditioned medium of Schwann cells (SC-CM) (Fig. 5d). The increased ZO-1 and decreased Vimentin expression in Panc-1-siVDAC1 cells were observed, indicating reduced EMT activity (Fig. 5e and f). Moreover, siVDAC1 reduced Panc-1 cell migration and invasion in SC-CM (Fig. 5g and h), which was further supported by wound healing assays showing decreased migration in Panc-1-siVDAC1 cells (Fig. 5i and j). In summary, *VDAC1* is highly expressed in PNI-positive PDAC tissues and is associated with PDAC cell proliferation, migration, invasion, and EMT in SC-CM, suggesting a key role for *VDAC1* in Schwann cell-mediated PDAC progression.

### Characterizing LUAD cell subpopulations of distinct malignancy and differential communications with CD8 T cells by C-Neural score

**Figure 6:**
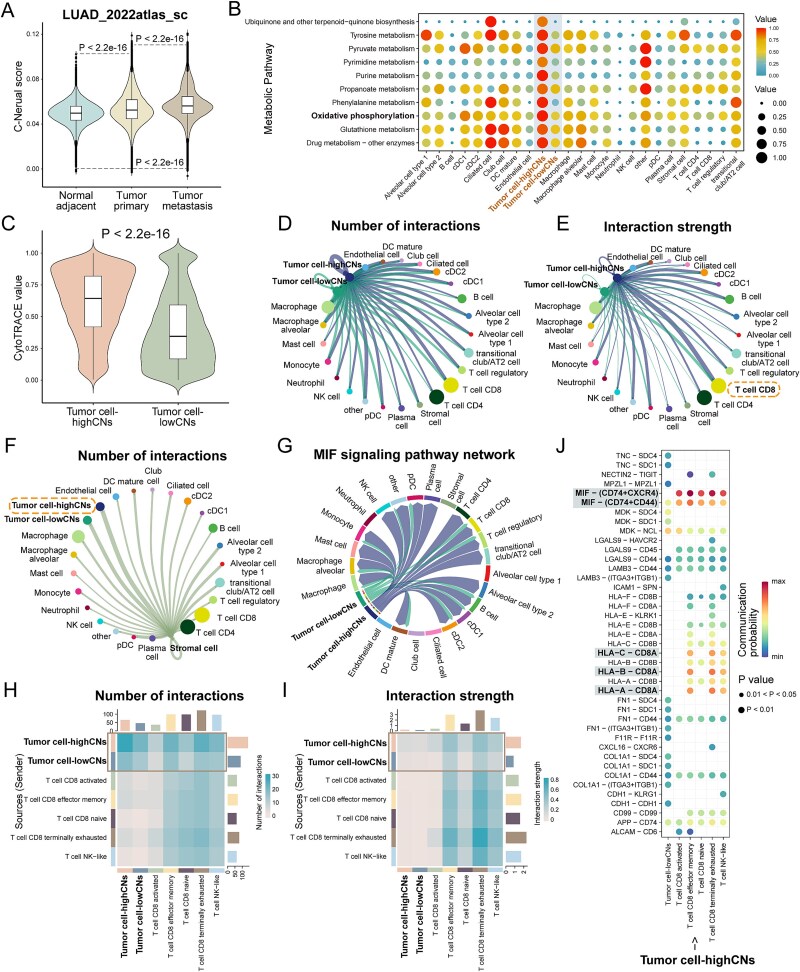
Comparison of tumor cells with discrepant C-Neural scores. (a) Distribution of C-Neural scores across cells from adjacent normal, tumor primary, and tumor metastasis samples in LUAD_2022atlas_sc dataset. (b) Differential metabolic pathways between tumor cell-highCNs and tumor cell-lowCNs. (c) Difference in cell differentiation values between tumor cell-highCNs and tumor cell-lowCNs. (d) and (e) Cell–cell communications among all cell types. The edge width of circle plot indicates the number or strength of cell–cell communications. (f) Cell–cell communications between stromal cell and other cell types. The edge width of circle plot indicates the number of cell–cell communications. (g) MIF signaling pathway communications among tumor cell-highCNs, tumor cell-lowCNs, and other cell subtypes. (h) and (i) Cell–cell communications among tumor cell-highCNs, tumor cell-lowCNs, and CD8 T subpopulations. The color of heatmap indicates the number or strength of cell–cell communications. (j) Significant ligand-receptor pairs contributing to the signaling from tumor cell-highCNs to CD8 T subpopulations. The dot color and size represent the calculated communication probability and *P*-values. *P*-values were calculated by one-sided Wilcoxon rank-sum test in (c) and one-sided permutation test in (j); *P* < .05 was considered statistically significant.

Dense neural innervation was observed in intact lung tissue, imaging by a whole-tissue immunolabeling and 3D assessment protocol, iDISCO(ace) [26]. We hypothesized that distal neural signals influence tumor cells in the TME. C-Neural scores were gradually increase in adjacent normal samples, primary samples, and metastatic samples in LUAD_2022atlas_sc dataset (*P* < 2.2e-16; Fig. 6a). Tumor cells exhibited higher C-Neural scores among all cell types (Fig. S15A). Significantly upregulated DEGs in tumor cell-highCNs were significantly enriched in OXPHOS pathway and neurodegenerative diseases (Fig. S15B). Additiona lly, purine metabolism and several metabolic processes were upregulated in tumor cell-highCNs (Fig. 6b). Tumor cell-highCNs also displayed lower differentiation (*P* < 2.2e-16; Fig. 6c; Fig. S15C). Altogether, the results indicated the higher malignancy of tumor cell-highCNs than tumor cell-lowCNs.

Tumor cell-highCNs sent stronger communications to CD8 T cells and received more communications from stromal cells (Fig. 6d-f; Fig. S16A and B). Tumor cell-highCNs interacted with other cell types via MIF signaling pathway (*P* < .01; Fig. 6g; Fig. S16C–E). We further investigated the cell–cell communications among tumor cells and CD8 T-cell subpopulations. Tumor cell-highCNs specifically interacted with effector memory CD8 T cells and terminally exhausted CD8 T cells (Fig. 6h and i; Fig. S16F and G). In addition, tumor cell-highCNs closely communicated with CD8 T cells through MIF and human leukocyte antigen (HLA)-related ligand-receptor pairs, and CD8 T cell sent signals to tumor cell-highCNs via CD99–CD99 and ITGB2–ICAM1 interactions (*P* < .01; Fig. 6j; Fig. S16H).

We noted that *VDAC1* was expressed in epithelial cells and macrophages in LUAD and NSCLC scRNA-seq datasets, with a significant positive correlation with tumor stage (Figs S17 and S18A). We then established an *in vitro* co-culture system between Schwann cells and LUAD cell line A549 with siVDAC1 or siNC. A549-siVDAC1 cells showed reduced cell density in SC-CM (Fig. S18B). Additionally, A549-siVDAC1 exhibited increased ZO-1 expression and decreased Vimentin expression in SC-CM (Fig. S18C and D). Migration and invasion were also significantly reduced in A549-siVDAC1 cells co-cultured with Schwann cells (Fig. S18E-H). These findings, along with the results from the PAAD co-culture system, suggest that *VDAC1* likely mediates the effects of Schwann cells on cancer progression.

### Patients responding to immunotherapy response have elevated C-Neural scores

**Figure 7:**
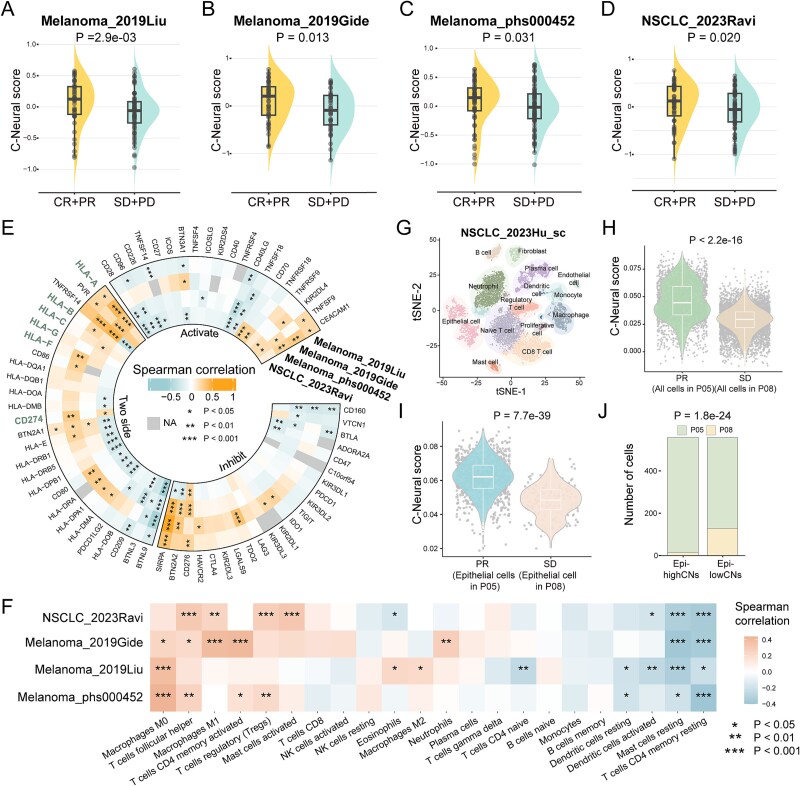
Higher C-Neural score indicates response to immunotherapy. (a)–(d) Distribution of C-Neural scores between responsive [complete response (CR) and partial response (PR)] and non-responsive [stable disease (SD) and progressive disease (PD)] patients in melanoma and NSCLC immunotherapy datasets. (e) Correlation between C-Neural scores and the expression of immune checkpoint genes in melanoma and NSCLC immunotherapy datasets. (f) Correlation between C-Neural scores and infiltration of immune cells in immunotherapy datasets. (g) Clusters identified and annotated into 14 cell types for three immunotherapy pre-treatment samples in NSCLC_2023Hu_sc dataset. (h) Distribution of C-Neural scores between all cells of responsive patient (PR, BD_immune05) and non-responsive patient (SD, BD_immune08). (i) Distribution of C-Neural scores between epithelial cells of responsive patient and non-responsive patient. (j) Differential distribution of responsive cells and non-responsive cells between high and low C-Neural score groups. *P*-values were calculated by one-sided Wilcoxon rank-sum test in (a)–(d), (h), and (i), Spearman’s rank correlation analysis in (f), and chi-square test in (j); *P* < .05 was considered statistically significant.

Given that tumor cell-highCNs communicate with CD8 T cells, we further investigated the relevance of C-Neural scores with immune cells infiltration and immunotherapy response (Fig. S1D). In bulk RNA-seq datasets of ICI pre-treatment melanoma, NSCLC, and BC samples, C-Neural scores were significantly higher in responsive patients compared to non-responsive patients (*P* < .1; Fig. 7a–d; Fig. S19A–C). The area under the curve (AUC) values of receiver operating characteristic curve were over 0.60 in all datasets (Fig. S19D–J). We compared the predicted performance of C-Neural score with other 10 signatures and algorithms. The AUC values of C-Neural scores were the highest in Melanoma_2019Liu, Melanoma_phs000452v3, Melanoma_2015VanAllen, and NSCLC_2023Ravi datasets (Fig. S20). Patients with high C-Neural scores were characterized by elevated mutations (Fig. S21A–D). We revealed positive associations of C-Neural scores with HLA and CD274 (Fig. 7e). Activated memory CD4 T cells, follicular helper T cells, M0 macrophages, and M1 macrophages were positively correlated with C-Neural scores, while resting memory CD4 T cells were significantly negatively correlated (*P* < .05; Fig. 7f; Fig. S21E). CD8 T cell infiltration was significantly positively correlated with C-Neural scores, which implied the increased interactions between epithelial cells and CD8 T cells in high C-Neural score samples, consistent with our previous analysis of LUAD_2022atlas_sc dataset.

We investigated the association between C-Neural score and immunotherapy response at single-cell resolution. Cells from responsive LUAD patients exhibited significantly higher C-Neural scores (*P* < 2.2e-16; Fig. 7g and h; Fig. S22A–D). Epithelial cells displayed higher C-Neural scores in responsive LUAD patient (*P* = 7.7e-39; Fig. 7i). Epithelial cells from non-responsive LUAD patient were predominantly distributed in low C-Neural score group (*P* = 1.8e-24; Fig. 7j). Additionally, C-Neural scores were higher in responsive NSCLC bulk samples (Fig. S22E). These comparative analyses underscored the association of elevated C-Neural scores with immunotherapy response, indicating the activation of antitumor immune cells.

### Prediction of anticancer drug treatment based on C-Neural score

**Figure 8:**
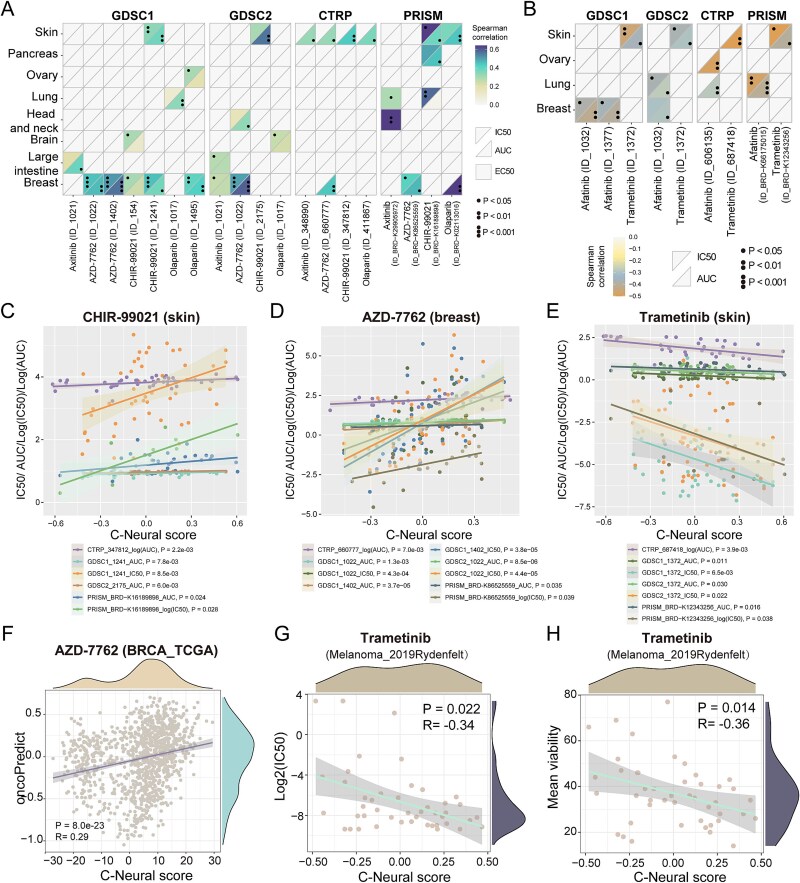
Prediction of drug therapy by C-Neural score. (a) Correlation between C-Neural scores and drug-response values (IC50, AUC, and EC50) for four drugs that were overlapped in genomics of drug sensitivity in cancer (GDSC, GDSC1 and GDSC2), cancer therapeutics response portal (CTRP), and dependency map (DepMap) portal (PRISM repurposing v19Q4) datasets. (b) Correlation between C-Neural scores and drug-response values (IC50 and AUC) for two drugs that were overlapped in four pharmacological screening datasets. The heatmap shows correlation values. (c) Significant positive correlation between drug-response values of CHIR-99021 and C-Neural scores in skin tissue. (d) Significant positive correlation between drug-response values of AZD-7762 and C-Neural scores in breast tissue. (e) Significant negative correlation between drug-response values of trametinib and C-Neural scores in skin tissue. (f) Significant positive correlation between oncoPredict values of AZD-7762 and C-neural scores in BRCA_TCGA. (g) Negative correlation between IC50 values of trametinib and C-Neural scores in Melanoma_2019Rydenfelt dataset. (h) Negative correlation between mean viability values of cell lines treated by trametinib and C-Neural scores. *P*-values were calculated by Spearman’s rank correlation analysis; *P* < .05 was considered statistically significant.

Targeting cancer hallmarks has become a rational approach for drug therapy, aiming to disrupt multiple functional capabilities of cancer. We investigated the potential of C-Neural scores to guide the selection of therapeutic drugs for cancer treatment. (Fig. S1E). We identified drugs whose dose–response values significantly correlated with C-Neural scores in cancer cell lines of 10 tissue types (Fig. S23A–E). In four pharmacological screening datasets, axitinib, AZD-7762, CHIR-99021, and olaparib were positively correlated with C-Neural scores, while afatinib and trametinib were negatively correlated with C-Neural scores (*P* < .05; Fig. 8a–e).

We next examined these observations using drug sensitivity values inferred by oncoPredict algorithm in TCGA dataset. Axitinib, AZD-7762, and olaparib were positively correlated with C-Neural scores, while afatinib and trametinib were negatively correlated with C-Neural scores (Fig. S23F and G). In BRCA_TCGA, the drug sensitivity values of AZD-7762 were positively correlated with C-Neural scores, suggest that BC patients with lower C-Neural scores may be sensitive to AZD-7762 (*P* = 8.0e-23, *R* = 0.29; Fig. 8f). The IC50 values of trametinib and cell viability were negatively correlated with C-Neural scores in melanoma cancer cell lines (*P* < .05; Fig. 8g and h). Melanoma cell lines with high C-Neural scores exhibited decreased IC50 values and viability (Fig. S23H and I). These results suggest that melanoma patients with higher C-Neural scores may benefit from trametinib, collectively indicate the potential of C-Neural score in predicting cancer drug therapy.

## Discussion

Neural infiltration, as an aberrant condition within the TME, holds the potential to be a novel cancer hallmark. Our study proposed C-Neural score for evaluating neural infiltration levels in multiple cancer types, revealing associations with cancer malignancy.

The nervous system communicates with cancer cells through modulation of components within the TME. Nerves serve as conduits for cancer spread and migration and provide increased survival advantage for cancer cells [7]. To comprehensively evaluate the neural effects in TME, we collected neural genes to propose a score for assessing C-Neural score across 10 cancer types. Notably, C-Neural scores were significantly elevated in PNI-positive cancer samples and Schwann cells enriched regions, demonstrating the predictive potential for nerve in TME.

Cancer hallmarks encompass essential biological capabilities driving malignant transformation and progression. C-Neural scores were positively correlated with tumor purity, tumor cell content, tumor size, stemness, recurrence, metastasis, poor outcome, and cancer hallmark scores in several cancer types. Moreover, epi-highCNs exhibited pronounced CNV and lower cell differentiation, suggesting the capacity of identifying malignant epithelial cells by C-Neural score at scRNA-seq resolution. Cancer cells require OXPHOS to strive, and we observed the upregulation of OXPHOS in epi-highCNs and tumor cell-highCNs. The findings emphasize that elevated C-Neural scores are linked to the acquisition of cancer hallmarks.

The PPI network between epi-highCNs and Schwann cells indicates strong connections through ECM and adhesion molecules [27]. Patients with low expression of gene pairs appear to have poor prognosis. Schwann cells may facilitate cancer progression through *VDAC1*. A previous study showed that Schwann cells-derived CXCL5 promotes EMT, invasiveness, and metastasis of lung cancer cells [28]. Schwann cells enhance PAAD cells aggressiveness through TGFβ-dependent manner [29]. Additionally, TNF-α levels increase in Schwann cells treated with different tumor cell lines or obtained from tumor-bearing animals [30]. We found *VDAC1* expression is significantly positive correlation with cytokines such as *CXCL5*, *TGFA*, and *TGFBR1* in multiple PAAD and LUAD datasets (Fig. S24). Additionally, *VDAC1* silencing significantly reduced TGF-β expression in LUAD cell line [31]. VDAC lies at the junction between cytoplasm and mitochondria and transports most anionic metabolites [32]. TGF-β1 increases mitochondrial oxygen consumption and ATP generation in the presence of diverse energy substrates [33]. In addition, terminal/perisynaptic Schwann cells release Ca2+ in response to synaptic activity [34]. VDAC1 may facilitate Ca2+ transport in cancer cells [35]. VDAC1 affects various immune cells, such as T cell metabolism and function, dysregulation of VDAC1 has been linked to autoimmune diseases [36]. These findings highlight potential cytokines and signaling pathways through which Schwann cells interact with *VDAC1*, emphasizing the need for further exploration by co-culture and *in vivo* experiments. Additionally, spatial multi-omics techniques can be utilized to spatially elucidate the signals among neural cells and other components in TME in future studies.

FN1 is located in the ECM of all cells as linear and branched networks that surround and connect neighboring cells [37]. We observed increased FN1 signaling from Schwann cells to epi-highCNs, indicating stronger connections. Additionally, tumor cell-highCNs potentially interacted with CD8 T cells via MIF and HLA related ligand-receptor pairs. The MIF–CD74 axis promotes tumor growth and an immunosuppressive milieu [38]. MIF also functions as a non-cognate ligand for chemokine receptors, resulting in activating monocytes [39]. Further exploration is needed to understand the function of the MIF axis and crosstalk with neural signal in TME.

We revealed a significant increase in C-Neural scores in immunotherapy responsive patients. C-Neural scores were positively correlated with expression of *CD274* (coding PD-L1) and HLA genes. The integrated 20 clinical trials indicate PD-L1 expression links to improve response to immunotherapy [40]. IHC assay for PD-L1 has the most FDA approvals as a companion diagnostic for ICIs in specific tumor types [41]. Moreover, higher HLA-I expression is an emerging predictor for long-term response to ICIs in metastatic NSCLC patients [42]. In addition, C-Neural scores were positively correlated with infiltration of some antitumor immune cells, suggesting potential mechanism that cancer patients with high C-Neural scores may respond to immunotherapy. A deeper exploring of specific immune cells that affected by neural signals warrants investigation.

Targeting neural signal is an emerging therapeutic strategy for cancers [43]. AZD-7762 is an ATP-competitive checkpoint kinase inhibitor, potentiates antitumor efficacy when combined with DNA-damaging agents [44]. High C-Neural score samples exhibited upregulation of DNA replication and repair proteins, possibly contributing to resistance mechanisms in BC patients. Trametinib is an ERK/MAPK inhibitor, historically used in BRAF-mutant melanoma and NSCLC and in NF1-related nervous system tumors recently [45]. ERK/MAPK signaling pathway is critical for neurodevelopment [46]. We supposed that trametinib may disadvantage neural growth in TME, potentially explaining its efficacy in melanoma patients with high C-Neural scores. Future pharmacodynamic experiments are needed to validate these predictions and explore the underlying mechanisms.

Currently, most researches concentrate on the prediction of PNI, whereas ignore the neural signals in the TME. For example, Park *et al*. [47] adopted a deep learning-based method U-Net to detect PNI regions. Kartasalo *et al*. [48] trained deep neural networks by biopsy cores for detection of PNI in prostate cancer. Additionally, a small fraction of work focused on assessing certain neural signal in cancer. Dai *et al.* [49] obtained presynapse genes from Syngo portal (https://www.syngoportal.org/) and evaluated the neural signal by averaging the expression of genes that expressed in distal nerve terminals. Different from the aforementioned studies, we evaluated the integrated neural infiltration levels in the TME across multiple cancer types based on transcriptome.

In conclusion, this study elucidates level of neural infiltration on TME, which has broad implications for our better understanding of the complex mechanisms of cancer neuroscience. As a potential new cancer hallmark, it enhances therapeutic strategies by providing more accurate patient stratification and treatment selection.

**Key Points**

- The C-Neural score systematically characterizes the level of neural infiltration, which is a potential new cancer hallmark in TME.
- Epithelial cells with high C-Neural scores exhibit highly malignancy and frequently communicate with Schwann cells in pancreatic ductal adenocarcinoma.
- Schwann cells may facilitate cancer progression through upregulation of *VDAC1*, which is primarily expressed in cancer cells.
- Melanoma and NSCLC patients who respond to immunotherapy exhibit elevated C-Neural scores, and melanoma patients with high C-Neural score could potentially benefit from treatment with trametinib.

## Supplementary Material

Supplementary_Materials_bbaf082

Table_S1_bbaf082

Table_S2_bbaf082

Table_S3_bbaf082

Table_S4_bbaf082

Table_S5_bbaf082

Table_S6_bbaf082

Table_S7_bbaf082

Table_S8_bbaf082

Table_S9_bbaf082

Table_S10_bbaf082

## Data Availability

No new data were generated or analyzed in support of this research. All data analyzed during this study can be downloaded from public data resources. Other information was provided in the Supplementary Files.
