# Supplementary material for: Unveiling a novel cancer hallmark by evaluation of neural infiltration in cancer
Source: Brief Bioinform. 2025 Mar 7;26(2):bbaf082. doi: 10.1093/bib/bbaf082 (PMC11886572; doi:10.1093/bib/bbaf082)
Supplement: Supplementary_Materials_bbaf082 [file supplementary_materials_bbaf082.pdf]

Unveiling a Novel Cancer Hallmark by Evaluation of Neural Infiltration in Cancer

Contents

1. Supplemental Methods 4

1.1 Pan-cancer bulk RNA-seq datasets 4

1.2 Identification of cancer-specific differentially expressed neural genes 5

1.3 Evaluation of neural infiltration 5

1.4 Association of C-Neural score with cancer hallmarks and clinical characteristics 6

1.5 Functional enrichment analysis 7

1.6 Correlation analysis between C-Neural score and TME components 8

1.7 Survival analysis 9

1.8 Pan-cancer scRNA-seq data processing 9

1.9 Pancreatic cancer scRNA-seq processing 10

1.10 Non-small cell lung cancer scRNA-seq processing 12

1.11 Distribution of C-Neural score in pan-cancer scRNA-seq datasets 13

1.12 Cell subpopulations analysis 13

1.13 Cell-cell communication analysis 14

1.14 Cell culture 15

1.15 Co-culture system and conditioned medium collection 15

1.16 Cell proliferation assays 16

1.17 Wound healing assay 16

1.18 Transwell assay 16

1.19 Western blot 17

1.20 Immunofluorescence staining 17

1.21 Immunohistochemical staining 18

1.22 Association of C-Neural score with immunotherapy response 18

1.23 Prediction of drug treatment 19

1.24 Statistics 20

2. Supplemental Figures 22

Figure S1. Workflow of the study. 22

Figure S2. Number of DEG-Ns and correlation of C-Neural scores and cancer hallmarks. ...23

Figure S3. Functional enrichment analysis for C-Neural score-related differentially expressed genes. 24

Figure S4. C-Neural score correlate with perineural invasion and prognosis. 25

Figure S5. Correlation of C-Neural score with tumor microenvironment components. 26

Figure S6. Proportion of expressed DEG-Ns and distribution of C-Neural scores in spatial transcriptomics. 27

Figure S7. Cell annotation in PDAC_2023Xue_sc dataset. 28

Figure S8. Cell annotation in PAAD_2024Kim_sc dataset. 29

Figure S9. Cell-cell communications among cell types in PAAD scRNA-seq and snRNA-seq datasets. 31

Figure S10. Cell-cell communications among cell types in PAAD_2024Kim_sc dataset. 33

Figure S11. Significant ligand-receptor interactions in PAAD scRNA-seq and snRNA-seq datasets. 34

Figure S12. Cell-cell communications of FN1 signaling pathway among cell types in PAAD scRNA-seq and snRNA-seq datasets. 36

Figure S13. Protein-protein interactions between Schwann cells and epi-highCNs related to poor prognosis. 37

Figure S14. Expression of *VDAC1* among cell types in PAAD scRNA-seq datasets. 39

Figure S15. Evaluation of neural signal in LUAD scRNA-seq dataset. 41

Figure S16. Analysis of cell-cell communications in LUAD scRNA-seq dataset. 42

Figure S17. Expression of VDAC1 among cell types in LUAD and NSCLC scRNA-seq datasets. 44

Figure S18. Schwann cells promote LUAD progression mediate by VDAC1. 45

Figure S19. Prediction of immunotherapy response by C-Neural score. 46

Figure S20. Performance of C-Neural scores compared with other transcriptomic signatures or algorithms. 47

Figure S21. Correlation of C-Neural score and tumor mutational burden and immune infiltration. 48

Figure S22. Cell type annotation in NSCLC immunotherapy scRNA-seq data. 49

Figure S23. Identification of drugs for cancer based on pharmacological screening datasets.50

Figure S24. Correlation analysis for expression of *VDAC1* and cytokines. 51

3. Supplemental Tables 52

Table S1. Datasets for identifing differentially expressed neural genes. 52

Table S2. Source of neural genes. 52

Table S3. Neural genes. 52

Table S4. Cancer-specific differentially expressed neural genes. 52

Table S5. Independent validation datasets. 52

Table S6 Spearman correlation between stage/grade and C-Neural scores. 52

Table S7. Pan-cancer scRNA-seq datasets. 52

Table S8. Datasets for melanoma and NSCLC by immunotherapy. 52

Table S9. The formulas of transcriptomic ICI signatures or algorithms from published literatures. 52

Table S10. Pharmacological screening data for cancer cell lines. 53

4. Abbreviations 54

5. References 56

1. Supplemental Methods

1.1 Pan-cancer bulk RNA-seq datasets

Normalized gene expression profiles for ten cancer types (lung adenocarcinoma [LUAD], colorectal cancer [CRC], glioma, breast cancer [BC], prostate cancer [PCa], Head and neck squamous cell carcinoma [HNSC], skin cutaneous melanoma [SKCM], renal cell carcinoma [RCC], pancreatic adenocarcinoma [PAAD], and ovarian cancer [OV]) with matching normal samples were obtained from The Cancer Genome Atlas (TCGA, https://portal.gdc.cancer.gov/), Gene Expression Omnibus (GEO, https://www.ncbi.nlm.nih.gov/gds/), ArrayExpress (https://www.ebi.ac.uk/biostudies/arrayexpress), and The Genotype-Tissue Expression (GTEx, https://www.gtexportal.org/home/) data portals (Table S1). The gene expression and clinical data of TCGA and GTEx were downloaded from UCSC Xena (https://xenabrowser.net/datapages/). A total of 40 expression profiles for cancer and normal samples were collected, in which number of samples were more than 30, therein four expression profiles for each cancer type.

The transcriptome data of brain, skin, pancreas, and ovary normal tissues in GTEx were used, ensuring RNA integrity (RNI) numbers were greater than seven. We obtained raw expression profiles by Affymetrix platform chip (HumanGeneChip HG-U133 Plus 2.0 arrays) for 170 glioma samples (E-MTAB-3892), and used R package “affy” (v1.74.0) to normalized expression value based on the Robust Multichip Average (RMA) method. The count values for 148 paired prostate cancer samples were downloaded from GEO (GSE229904), the count values were

normalized to the Transcripts Per Million (TPM) values.

1.2 Identification of cancer-specific differentially expressed neural genes

The neural genes were primarily collected from the following four sources (Table S2): (1) Ten pathways related to nervous system or neural signal transmission from the Kyoto Encyclopedia of Genes and Genomes (KEGG, https://www.genome.jp/kegg/). (2) The components involving signal reception and transmission of neurons, neurotransmitters, or neurotrophins from Gene Ontology (GO, https://geneontology.org/), including 60 GO terms (Molecular Function, Cellular Component, and Biological Process) downloaded from The Molecular Signatures Database (MSigDB, https://www.gsea-msigdb.org/gsea/msigdb/). (3) Neuronal cell marker genes from the literatures. (4) Perineural invasion (PNI)-related genes from the literatures. After removing duplicates, 1,889 neural genes were retained (Table S3).

For each paired gene expression profiles, one-sided Wilcoxon rank sum-test was used to identify significantly differentially expressed neural genes (DEG-Ns) ($P < 0.01$). Within each of the four datasets per cancer type, a gene was categorized as an upregulated DEG-N (DEG-Nup) in tumor samples if it exhibited upregulation in at least two datasets and non-significant downregulation in the remaining datasets. Conversely, a gene was categorized as a downregulated DEG-N (DEG-Ndown) (Table S4).

1.3 Evaluation of neural infiltration

For bulk RNA-seq data, we computed the C-Neural score using Gene Set Variation

Analysis (GSVA) method with R package "GSVA" (v1.44.5). The C-Neural score for a sample was determined by subtracting the GSVA value for DEG-Ndown from the GSVA value for DEG-Nup.

$$C\text{-}Neural\ score = GSVA_{\ (DEG\text{-}Nup)} - GSVA_{\ (DEG\text{-}Ndown)}$$

For scRNA-seq data, we evaluated the C-Neural score using the UCell algorithm with the R package "UCell" (v2.4.0). The "maxRank" parameter was set as the top 5% expressed genes in the dataset. Similarly, the C-Neural score for a cell was calculated by subtracting the UCell value for DEG-Ndown from the UCell value for DEG-Nup. Ucell algorithm neglects the missing genes and expression values when calculate scores.

$$C\text{-}Neural\ score = UCell_{\ (DEG\text{-}Nup)} - UCell_{\ (DEG\text{-}Ndown)}$$

Samples were grouped into high C-Neural score group and low C-Neural score group according to the median in a dataset.

1.4 Association of C-Neural score with cancer hallmarks and clinical characteristics

Definition of cancer hallmark was refer from Hanahan *et al.*[1-3]. Cancer hallmark gene sets were collected from Catalogue of Somatic Mutations in Cancer (COSMIC, https://cancer.sanger.ac.uk/cosmic) database, The Cell Senescence Gene Database (CellAge, https://genomics.senescence.info/cells/), Chen *et al.*,(33823788) GO database, StemChecker (http://stemchecker.sysbiolab.eu) database, CR2Cancer (http://cis.hku.hk/CR2Cancer) database, and Host-Pathogen Interaction Database (HPIDB, https://cales.arizona.edu/hpidb/). We retained genes with Integrated

Stemness Signature score (ISS_OverallScore) > 4 in StemChecker, and retained genes with confidence score > 0.6 in HPIDB. The GSVA method was used to evaluate the cancer hallmark scores for TCGA samples.

Gene expression data and clinical information for nine cancer types of 31 datasets were obtained from TCGA, GEO, ArrayExpress, cBioPortal, Soltis *et al.*, and Gillette *et al.* (Table S5).[4, 5] Spearman's rank correlation analysis was used to assess the correlation between C-Neural score and tumor stage/grade in TCGA and independent datasets (Table S6). In HNSC_TCGA dataset, we assessed the difference in C-Neural score between PNI-positive and PNI-negative tumor samples. In COAD_TCGA dataset, we assessed the difference in C-Neural score between PNI-positive and PNI-negative advanced (Stage IV) tumor samples. The PNI information of partial LUAD_TCGA samples were collated from the pathology report text from Kefeli *et al.*[6] We assessed the difference in C-Neural score between PNI-positive and PNI-negative alive LUAD patients.

We also compared the C-Neural score between primary and recurrent samples, as well as primary and metastatic samples in LUAD, glioma, SKCM, and RCC datasets. Tumor TNM stage, stage or grade was quantified, and the correlation with the C-Neural scores was assessed using Spearman's rank correlation analysis.

1.5 Functional enrichment analysis

For each cancer type in TCGA, samples were grouped into high and low C-Neural score groups. R package "limma" (v3.54.2) was used to normalized count values by "voom" method, and to identify significantly differentially expressed genes (DEGs)

with $P < 0.05$ and $|\log FC| > 1$.

The KEGG PATHWAY and KEGG BRITE terms were obtained from KEGG database. A hypergeometric distribution model was used to identify significantly enriched terms ($P < 0.05$).

1.6 Correlation analysis between C-Neural score and TME components

The fraction of stromal, immune, and tumor components inferred using Estimation of STromal and Immune cells in MAlignant Tumours using Expression data (ESTIMATE) method for LUAD samples was obtained from Soltis *et al.* and Gillette *et al.* Additionally, Gillette *et al.* provided the evaluation of tumor purity by TSNet method and stemness index calculated by Malta *et al.*[7, 8]

The abundances of 22 immune cells were estimated using the Cell-type Identification By Estimating Relative Subsets Of RNA Transcripts (CIBERSORT) method.[9] We utilized the gene signature matrix (LM22) from CIBERSORT (https://cibersortx.stanford.edu/cshome.php) website and R package "CIBERSORT" (v1.03) to compute the proportions of infiltrating immune cells in tumor samplers. CIBERSORT values for TCGA samples were downloaded from the TIMER2.0 (http://timer.comp-genomics.org/timer/) website. Spearman's rank correlation analysis was used to assess the correlation between C-Neural scores and immune cell infiltration.

Tumor purity assessments for TCGA samples using five methods were obtained from Aran *et al.*[10] : (1) ESTIMATE; (2) ABSOLUTE; (3) Leukocytes unmethylation for purity (LUMP); (4) Immunohistochemistry (IHC); (5) Consensus

measurement of purity estimations (CPE), which represents the median purity level after normalizing estimates from the aforementioned four methods. Spearman's rank correlation analysis was used to evaluate the correlation between C-Neural scores and tumor purity values. Additionally, tumor purity, tumor size, and tumor cell content measures were obtained from other independent datasets (Table S5).

1.7 Survival analysis

High-quality survival outcome data from TCGA were obtained from Liu *et al.*,[11] including four major clinical outcome endpoints: (1) overall survival (OS) event and OS time; (2) disease-specific survival (DSS) event and DSS time; (3) disease-free interval (DFI) event and DFI time; (4) progression-free interval (PFI) event and PFI time. We also obtained the OS, progression-free survival (PFS), metastasis-free survival (MFS), and relapse free survival (RFS) data from other datasets (Table S5). Samples were stratified based on the median of C-Neural scores, and differences in survival outcomes were assessed using the log-rank test. Kaplan-Meier plots utilized to visualize the results. Survival analysis was conducted using R packages "survival" (v3.5.7) and "survminer" (v0.4.9).

1.8 Pan-cancer scRNA-seq data processing

A total of 55 scRNA-seq datasets for ten cancer types were obtained from GEO, Synapse (https://www.synapse.org/#), Genome Sequence Archive (GSA, https://ngdc.cncb.ac.cn/gsa/), and Qian *et al.* (Table S7). The scRNA-seq data from each study was separately processed using R package "Seurat" (v 4.2.1). For cell-quality filtering, cells with < 200 expressed genes, > 6 median absolute

deviations of expressed genes, and > 20% mitochondrial transcripts were excluded. The count values were log-normalized with a scale factor of 10,000. The top 2,000 variably expressed genes were identified using the FindVariableFeatures function. The ScaleData function were used to regress out unwanted sources of variation. Dimensionality was reduced via principal component analysis (PCA) incorporating highly variable features. To remove batch effects while preserving biological variation, R package "harmony" (v1.2.0) was used to integrate cells across individuals.[12] Cell clusters were identified using the first 30 PCs with a clustering resolution of 0.8 via the FindClusters function. Cluster-specific genes were identified using the FindAllMarkers function, and the clusters were annotated by comparing these genes with canonical cell markers.

1.9 Pancreatic cancer scRNA-seq processing

The scRNA-seq from four pancreatic ductal adenocarcinoma (PDAC) tissues with Schwann cells accumulation was obtained from Xue *et al.*, referred to as "PDAC_2023Xue_sc" dataset.[13] The scRNA-seq data was processed using Seurat method. Cells containing > 200 expressed genes and mitochondrial UMI rates < 40% passed the cell quality filtering, mitochondrial genes were subsequently removed. Following log-normalized, the top 2,000 variably expressed genes were identified for PCA. Batch effects among samples were mitigated using the Harmony method. Cell clusters were identified using the first 30 PCs with a resolution of 0.8, and visualized using the t-distributed Stochastic Neighbor Embedding (t-SNE). Clusters were annotated by comparing the cluster-specific genes with canonical cell markers.

Fibroblasts, also known as cancer-associated fibroblasts (CAF) in the TME, were classified into three subtypes: myofibroblastic CAF (myCAF), inflammatory CAF (iCAF), and reticular like CAF (r-lCAF), based on cell markers summarized by Cords *et al.*[14] The "Epithelial cell/Ductal cell" population, from which pancreatic ductal adenocarcinoma (PDAC) typically originates, were grouped into high C-Neural score (epi-highCNs) population and low C-Neural score (epi-lowCNs) population according the median of C-Neural score.

The non-immune scRNA-seq data of 17 PAAD patients (13 PDAC and 4 intraductal papillary mucinous neoplasms with high-grade dysplasia or invasive carcinoma tissues) (GSE194247) were obtained from Kim *et al.,* referred to as "PAAD_2024Kim_sc" dataset.[15] R package "Seurat" was used to process the scRNA-seq data. Cells with detected genes from 500 to 7,000, UMI counts > 2,000, and percentage of mitochondrial genes < 10% were reserved for analysis. Following log-normalized, the top 2,000 variably expressed genes were identified for PCA. Batch effects among samples were mitigated by Harmony method. Cell clusters were identified using the first 30 PCs with a resolution of 0.3, and visualized using the Uniform Manifold Approximation and Projection (UMAP) method. Clusters were annotated by comparing the cluster-specific genes with canonical cell markers. The "Epithelial/Ductal cell" population were grouped into epi-highCNs and epi-lowCNs populations according the median of C-Neural score. The spatial transcriptome data of patient "SS1960050" were accessed with the accession number GSE235315. R package "Seurat" was used to process the data. R package "UCell" was used to

calculate C-Neural scores for spots. Spots were identified by the normalized expression values of Schwann cell markers (*SOX10*, *S100B*, and *NGFR*): (1) All markers were more than 1. (2) At least two of the three markers were more than 1; (3) At least one of the three markers were more than 1. The the C-Neural score between high expressed spots and other spots were compared.

The single-nucleus RNA sequencing (snRNA-seq) data for 15 PDAC samples without neoadjuvant treatment from Hwang *et al.* were downloaded from the Single Cell Portal at https://singlecell.broadinstitute.org/single_cell/study/SCP1089, which we named as "PDAC_2022Hwang_sn" dataset.[16] Tumor cells were grouped into high C-Neural score population (tumor cell-highCNs) and low C-Neural score population (tumor cell-lowCNs) population.

1.10 Non-small cell lung cancer scRNA-seq processing

Salcher *et al.* aggregated 29 publicly available datasets to construct a non-small cell lung cancer (NSCLC) single-cell atlas, comprising 556 samples from 318 patients.[17] We downloaded the scRNA-seq data with cell type annotation information from Zenodo (https://doi.org/10.5281/zenodo.6411867), and extracted 410,927 cells from 156 LUAD samples, designated as the "LUAD_2022atlas_sc" dataset. Tumor cells were grouped into 11,709 tumor cell-highCNs and 11,708 tumor cell-lowCNs based on the median score. The FindMarkers function in Seurat was used to identify significantly upregulated DEGs in tumor cell-highCNs compared to tumor cell-lowCNs with logFC > 0.25, P value < 0.05, and minimum percentage (min.pct) > 0.1.

1.11 Distribution of C-Neural score in pan-cancer scRNA-seq datasets

For each scRNA-seq dataset, we calculated the C-Neural score for all cells and grouped them based on the median score. For each cell type in a dataset, we computed the distribution proportion in high C-Neural score population (P-high$_t$) and low C-Neural score population (P-low$_t$). We calculated the foldchange (FC) value between P-high$_t$ and P-low$_t$. For cell types annotated in multiple datasets, we obtained multiple P-high$_t$ values and P-low$_t$ values. One-sided Wilcoxon rank-sum test was executed to assess difference in the distribution of P-high$_t$ values and P-low$_t$ values.

1.12 Cell subpopulations analysis

To detect malignant cancer cells, we performed Copy number Karyotyping of Tumors (CopyKAT) algorithm to estimate single-cell copy number variation (CNV) landscapes using scRNA-seq by R package "copykat" (v1.1.0).[18] Epithelial cells were defined as aneuploid (malignant) and diploid (non-malignant) cells. The chi-squared test was used to evaluate the distributional difference of aneuploid and diploid cells between epi-highCNs and epi-lowCNs.

The Cellular (Cyto) Trajectory Reconstruction Analysis using gene Counts and Expression (CytoTRACE) algorithm was used to estimate the differentiation (stemness) status of epithelial cells or tumor cells using R package "CytoTRACE" (v0.3.3).[19] Cells were given a CytoTRACE score indicating differentiation potential, with higher score signifying poor differentiation. The Slingshot algorithm was used to infer cells lineage and pseudotime via R package "slingshot" (v2.8.0).[20] Slingshot identified the lineage structure of epithelial cells based on clustering-based minimum

spanning tree, and utilized simultaneous principal curve method to fit branch curve to the lineage.

scMetabolism is a computational pipeline for quantifying single-cell metabolism.[21] We utilized R package "scMetabolism" (v0.2.1) to evaluate the metabolic pathway activity of epithelial cells or tumor cells based on AUCell quantification method.[22]

Epithelial-mesenchymal transition (EMT) genes were sourced from the EMTome (http://www.emtome.org/) database. We evaluated the EMT score for epithelial cells using R package "AUCell" (v1.22.0) based on the expression of 814 EMT genes, selecting the top 5% of genes in the dataset for the calculation.

1.13 Cell-cell communication analysis

Cell-cell communications were inferred using R package "CellChat" (v1.6.1).[23] We focused on the human database in CellChat, identified over-expressed ligands or receptors by identifyOverExpressedGenes and identifyOverExpressedInteractions functions. Gene expression data were mapped onto a protein-protein interaction (PPI) network using the projectData function. Communication probabilities and cellular communication networks were computed and inferred using the computeCommunProb and filterCommunication functions (min.cells = 10). The computeCommunProbPathway and aggregateNet functions were used to infer the cell-cell communication at the signaling pathway level between each cell type. CellChat infers significant cell-cell communication by assigning probability values to each interaction and performing permutation tests. In addition, the statistical

method-based cell-cell communication were inferred using CellPhoneDB v5.0 by Python (v 3.10.7) [24].

We downloaded PPIs from Pathway Commons (https://www.pathwaycommons.org), an integrated resource of publicly available physical interactions involving proteins, DNA, RNA, and small molecules. The FindAllMarkers function in Seurat was used to identify marker genes of epi-highCNs and Schwann cells with logfc.threshold = 0.8, min.pct = 0.1, and p_val_adj = 0.05. The PPIs of marker genes were extracted from Pathway Commons to generate a PPI network. CellChat was then applied to identify significant PPIs between epi-highCNs cells and Schwann cells.

1.14 Cell culture

All cell lines were maintained at 37°C with 5% CO_2. The human PAAD cell line Panc-1 (CL-0184) and Schwann cell line sNF96.2 (CL-0864) were purchased from Wuhan Pricella Biotechnology Co., Ltd. The human LUAD cancer cell line A549 were purchased from the Chinese Academy of Sciences. sNF96.2 and Panc-1 cells were cultured in DMEM medium (Biological Industries, Israel) containing 10% fetal bovine serum (Biological Industries, Israel), and 100 U/ml penicillin, and 100 U/ml streptomycin. A549 cells were cultured in RPMI 1640 medium(Biological Industries, Israel) containing 10% fetal bovine serum. siVDAC1/siNC (negative control) was constructed by Genecreate Biological Co., Ltd (Wuhan, China). Transfection was carried out using Lipo2000 (Invitrogen, CA) by the manufacturer's recommendations.

1.15 Co-culture system and conditioned medium collection

For the co-culture assay, we used a 0.4-μm pore Transwell chamber (Corning, USA). After transfection of siVDAC1/siNC for 24 hours in Panc-1/A549, digested Panc-1/A549 cells (4×10^5) were seeded into the upper Transwell membrane. Then, 4×10^5 sNF96.2 cells were plated in the bottom chamber of 6-well plates. The cells were co-cultured for 48 hours and were used for future study. To collect the conditioned medium of sNF96.2 cells (SC-CM), cells were cultured in DMEM medium for 48 hours and the supernatant was centrifuged at 2000×g for 10 min to eliminate the cells and cell debris. All the SC-CM were used instantly or frozen at -80°C. For the incubation of Panc-1/A549 cells with SC-CM, Panc-1/A549 cells were incubated with SC-CM for 48 hours. Then, the cells were harvested for future assay.

1.16 Cell proliferation assays

To estimate the cell proliferation rate after co-culturing, Cell Counting Kit-8 (CCK-8) was used according to the manufacturer's instructions (Meilunbio, MA0218). Panc-1 and A549 cells were seeded in 96-well plates($1\text{-}5\times10^3$) after transfection, SC-CM was added for 48 hours and the optical density was measured at 450 nm (OD450).

1.17 Wound healing assay

Panc-1/A549 cells were seeded in a 6-well plate, transfected, and replaced with SC-CM culture. Monolayer fused cells scratched by the tip of a 200 μL micropipette. The wound areas were observed and photographed under a microscope (×20 objective) at 0, 24, and 48 hours after the scratch Image J was used to analyze the scratched images.

1.18 Transwell assay

To detect the migration and invasion ability of Panc-1 and A549 cells, a 24-well plate with Transwell membrane (8-μm, Corning, USA). For the invasion assay, inserts were pre-coated with Matrigel (354234,Corning, USA). Transfected Panc-1 and A549 cells were seeded into the upper chamber with a DMEM/RPMI 1640 medium containing 2% FBS. The bottom chambers were filled with SC-CM to stimulate upper chamber cells migration and invasion, the cells were co-incubated for 48h. Then, the cells were fixed with 4% paraformaldehyde and stained with 0.1% crystal violet (C0121, Beyotime, China). Stained cells were counted under a microscope (×20 objective). Image J software was used for cell number counting.

1.19 Western blot

Co-cultured cells' total protein was extracted by RIPA lysis buffer containing protease inhibitors (Beyotime, China). For western blotting, cell protein samples (50 μg) were separated with 10% SDS-PAGE gel electrophoresis and transferred to pure nitrocellulose membrane, and incubated with primary antibodies against ZO-1 (1:500, 21733-1-AP, Proteintech), Vimentin (1:500, 10366-1-AP, Proteintech), and GAPDH (1:500, 60004-1-Ig, Proteintech) overnight at 4°C. Western blot images were scanned by Odyssey Infrared Imaging System (Odyssey CLX, USA). Image Studio software was used to analyze the gray value of the target protein bands.

1.20 Immunofluorescence staining

For pancreatic cancer and adjacent tissues were collected from Harbin Medical University Cancer Hospital. Immunofluorescent staining was fixed with 4% paraformaldehyde then embedded in OCT (SAKURA, Finetek, Japan), and cut into

5-μm tissue sections. After acetone was fixed, hydrogen peroxide was blocked, and the tissue sections were incubated with anti-S100B (1:200, 66616-1-Ig, Proteintech) and anti-VDAC1 (1:200, 55259-1-AP, Proteintech). The images were captured by confocal microscope (FluoView FV10i, Olympus, Tokyo, Japan).

1.21 Immunohistochemical staining

Harbin Medical University Cancer Hospital provided PNI-positive and PNI-negative pancreatic cancer patients with paraffin-embedded sections. Praffin-embedded sections were incubated with 3% hydrogen peroxide solution to block peroxidase activity and co-incubated with anti-VDAC1(1:200, 55259-1-AP, Proteintech) at 4°C overnight after antigen repairing and blocking. The next day, tissues were incubated with secondary antibodies at room temperature for 1 h and visualized with DAB (ZSGB-BIO, Beijing, China). Five visual fields were randomly selected for evaluation in each section.

1.22 Association of C-Neural score with immunotherapy response

Bulk transcriptomic data from pre-treatment samples of four melanoma, two NSCLC and one BC immunotherapy cohorts were collected (Table S8). Additionally, we obtained scRNA-seq data from NSCLC patients undergoing anti-PD-1 treatment from GEO database (GSE207422), referred to as NSCLC_2023Hu_sc dataset [25]. We extracted scRNA-seq data from three pre-treatment NSCLC samples for analysis. The Seurat method was used to process scRNA-seq data. The expression matrix was log-normalized, and top 2,000 variably expressed genes were identified for PCA. The Harmony method was employed to remove batch effects. Cell clusters were identified

using the first 30 PCs with a clustering resolution of 0.5, visualized using t-SNE. Clusters were annotated by comparing cluster-specific genes with canonical cell markers.

The response to immune checkpoint inhibitors (ICIs) were evaluated by Response Evaluation Criteria in Solid Tumors (RECIST) standard [26]. Patients were grouped into response [complete response (CR) and partial response (PR) response] and non-response [stable disease (SD) and progressive disease (PD)] groups. In NSCLC_2023Hu_sc dataset, the response to anti-PD-1 treatment for lung squamous cell carcinoma (LUSC) patient BD_immune01 (P01) was not evaluated (NE), LUAD patient BD_immune05 (P05) had a PR, and LUAD patient BD_immune08 (P08) had a SD. The transcriptomic signatures and algorithms for predicting immunotherapy response were collected from published literatures (Table S9). The predictive effects were assessed by receiver operating characteristic (ROC) curve using R package "pROC" (v 1.18.5)

The immune checkpoint gene list was obtained from Hu *et al.*[27] Seventy-nine genes were categorized into "activate", "inhibit", and "two-side". Spearman's rank correlation analysis was used to evaluate the correlation between C-Neural scores and the expression of immune checkpoint genes.

1.23 Prediction of drug treatment

The pharmacological screening data were downloaded from multiple sources (Table S10): (1) Genomics of Drug Sensitivity in Cancer (GDSC, https://www.cancerrxgene.org/) (GDSC1 and GDSC2, v8.5); (2) Cancer Therapeutics

Response Portal (CTRP, https://portals.broadinstitute.org/ctrp.v2.1/) (v2.1); (3) Dependency Map (DepMap, https://depmap.org/portal/) portal (PRISM Repurposing v19Q4). Cell lines in CTRP and PRISM Repurposing were referenced from the Cancer Cell Line Encyclopedia (CCLE, https://depmap.org/portal/ccle/). Cell line information and normalized gene expression values were obtained from GDSC and DepMap (CCLE 2019). We collected half maximal inhibitory concentration (IC50), area under drug-response curve (AUC), and half maximal effective concentration (EC50) values of drugs. Within each tissue type, we identified drugs that IC50 values, AUC values, or EC50 values were significant positive or negative correlation with C-Neural score by Spearman's rank correlation analysis.

Drug sensitivity values for TCGA samples were inferred using the R package "oncoPredict" (v0.2) with GDSC2 as training data by calcPhenotype function. The identified drugs were validated by TCGA inferred drug sensitivity values. In addition, we obtained transcriptome data and drug sensitivity values of 49 melanoma cell lines from Rydenfelt *et al.*[28] We evaluated the correlation between C-Neural score and IC50 values or cell viability after Trametinib treatment.

1.24 Statistics

The one-sided Wilcoxon rank-sum test was applied for comparisons between two groups. The chi-square test was used to assess the categorical difference between groups. Spearman's rank correlation analysis was utilized to compute the association between two variables. Student's t test was used to compare the mean values of independent samples. Statistical analyses were conducted using R software

(https://www.r-project.org, v4.2.1) and GraphPad Prism software (https://www.graphpad.com/scientific-software/prism/, v10.0). $P < 0.05$ was considered statistically significant.

2. Supplemental Figures

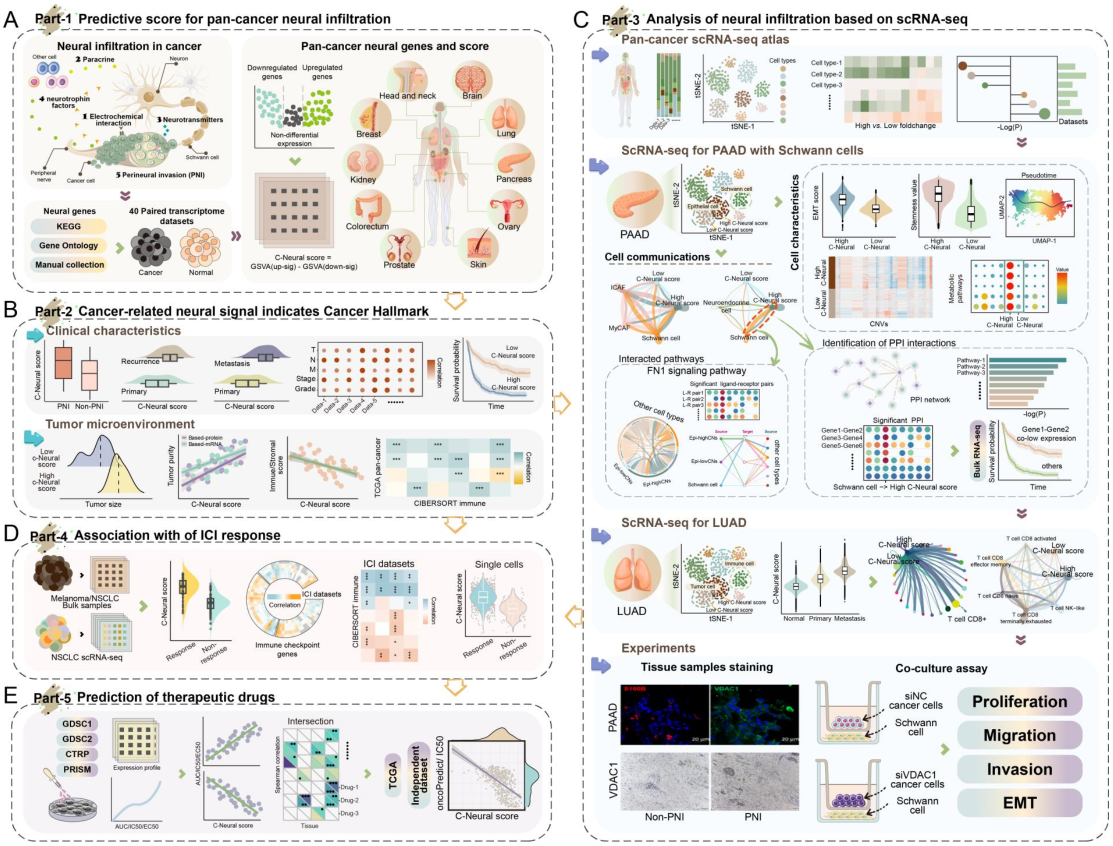

Figure S1. Workflow of the study.

(**A**) Collection of neural genes and evaluation of Cancer-related Neural infiltration (C-Neural score) for ten cancer types. **(B)** Analysis the correlation between C-Neural score and clinical characteristics and TME components. **(C)** Evaluation of neural infiltration across cell types using 55 scRNA-seq datasets. Epithelial cell subpopulations with discrepant neural signals were identified in PAAD and LUAD scRNA-seq datasets, with analysis of differences in cell characteristics and communications. Experiment validation for gene that mediates cancer progression by Schwann cells **(D)** Analysis of the correlation between C-Neural score and immunotherapy response based on bulk and scRNA-seq datasets. **(E)** Prediction of drug treatment for cancer based on C-Neural score and pharmacological screening data.

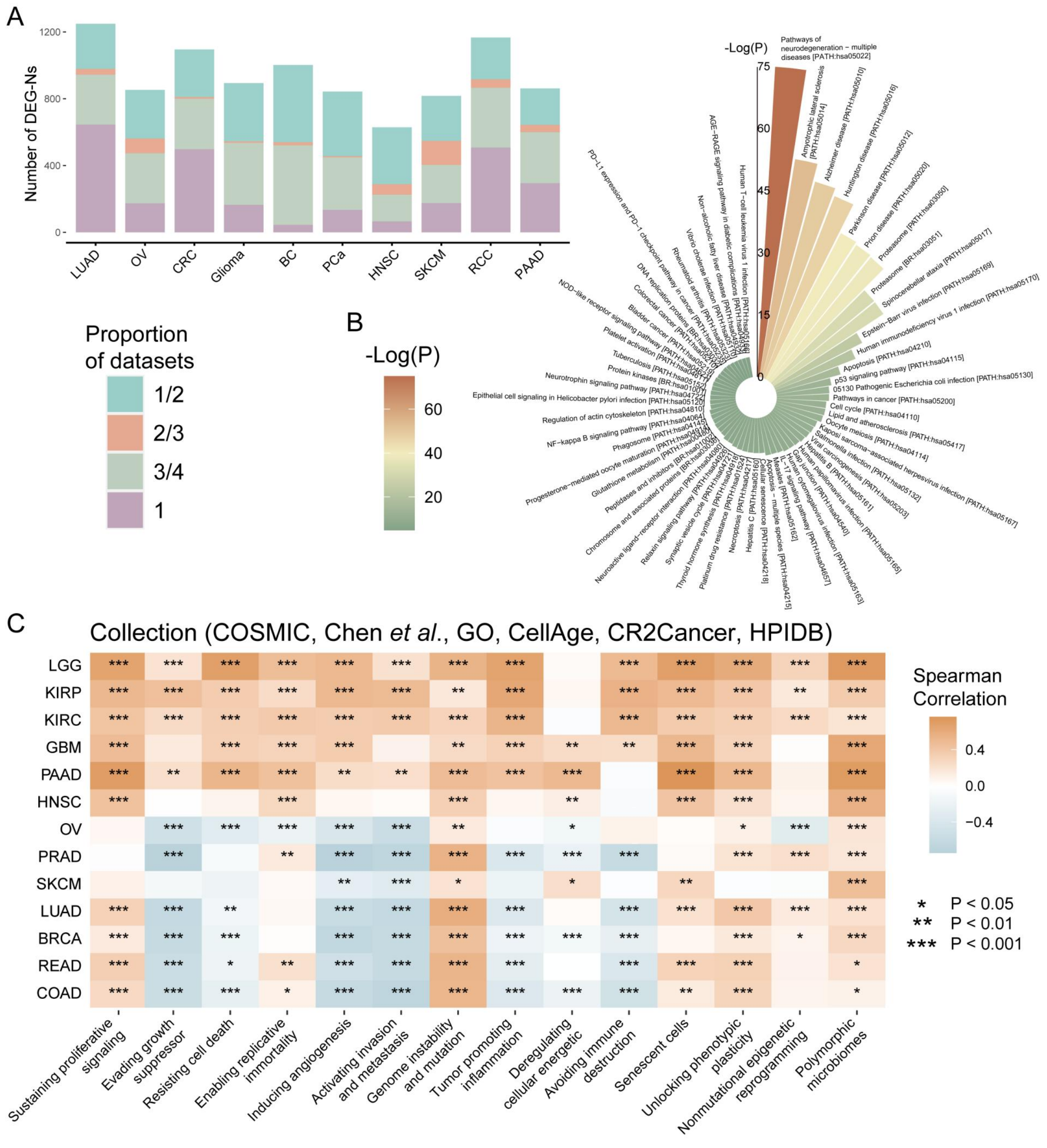

Figure S2. Number of DEG-Ns and correlation of C-Neural scores and cancer hallmarks.

(A) Number of DEG-Ns that were consistent identified based on different proportion of datasets within cancer types. (**B**) The KEGG functional enrichment analysis for cancer-specific neural genes that were upregulated in at least seven cancer types. **(C)** Correlation of C-Neural scores and cancer hallmark scores. *P* values were computed by hypergeometric test in **(B)** and Spearman's rank correlation analysis in **(C)**, *P* < 0.05 was considered statistically significant.

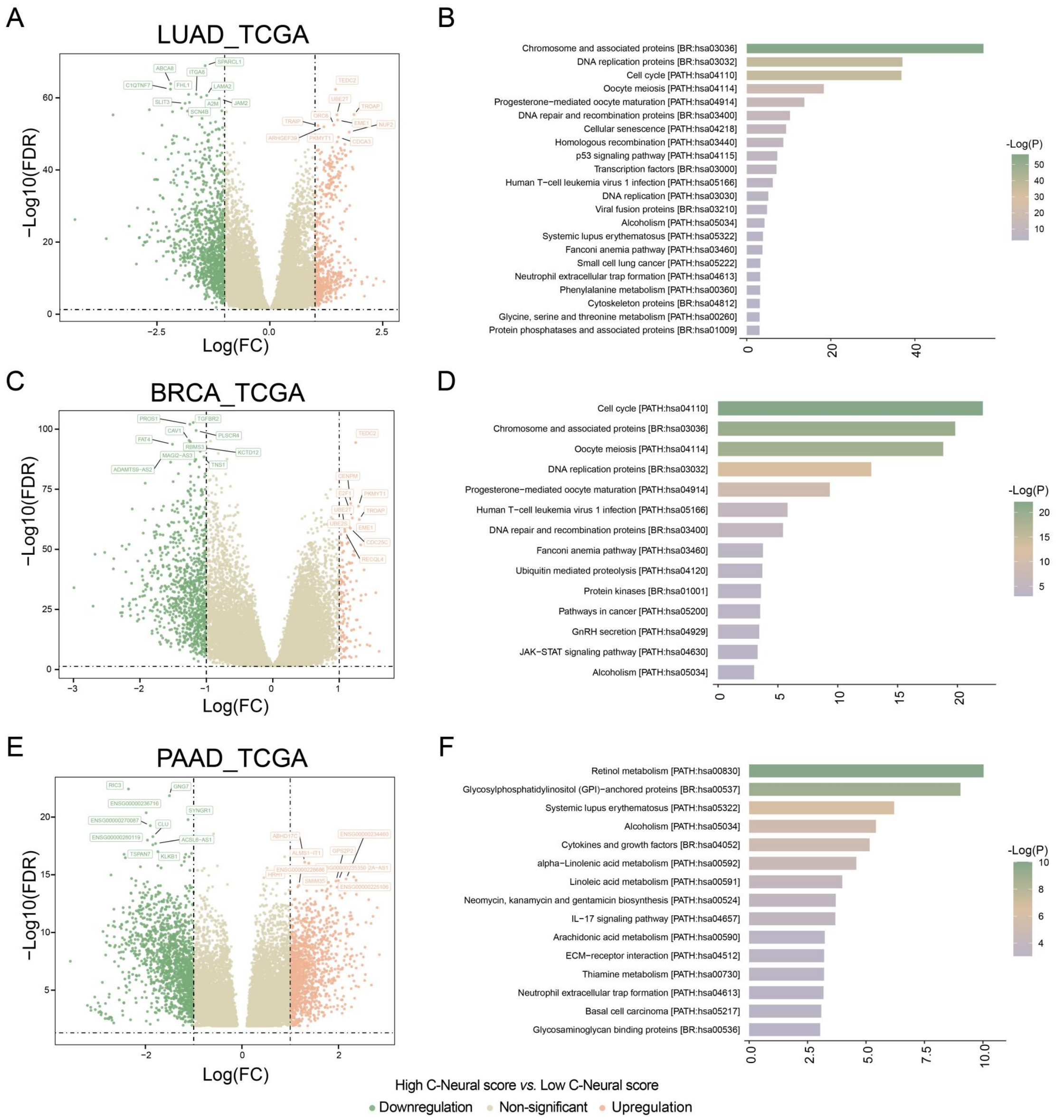

Figure S3. Functional enrichment analysis for C-Neural score-related differentially expressed genes.

(A), **(C)**, and **(E)** Patients were grouped based on the median C-Neural score, significantly DEGs were identified in LUAD, BRCA and PAAD of TCGA. The red dots represent upregulated genes, green dots represent downregulated genes. **(B)**, **(D)** and **(F)** The KEGG functional enrichment analysis for significantly upregulated DEGs in LUAD, BRCA and PAAD of TCGA. *P* values were computed by hypergeometric test in **(B)**, **(D)** and **(F)**, $P < 0.05$ was considered statistically significant.

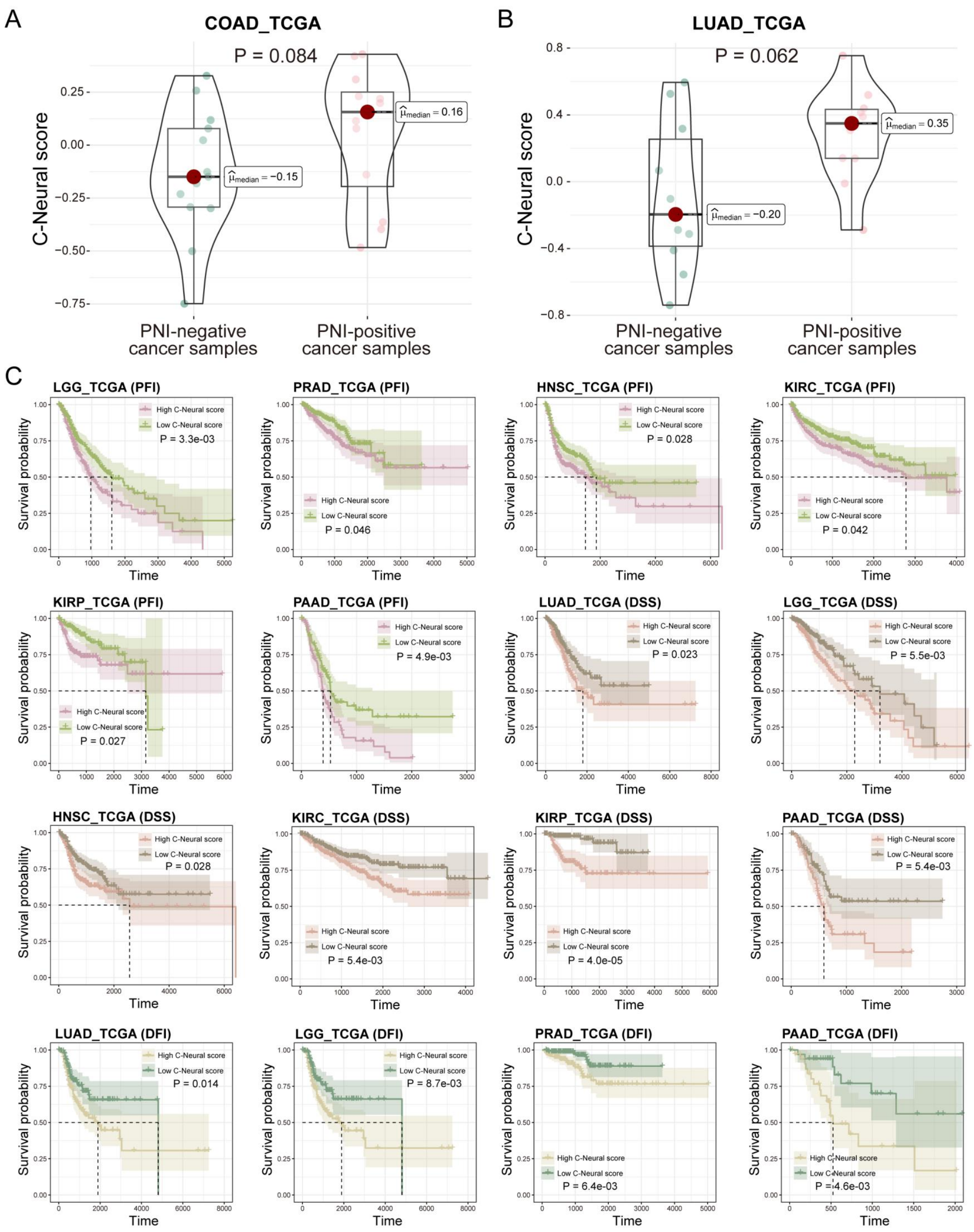

Figure S4. C-Neural score correlate with perineural invasion and prognosis.

(A) Distribution of C-Neural scores between the PNI-positive and PNI-negative advanced COAD samples. **(B)** Distribution of C-Neural scores between the PNI-positive and PNI-negative alive LUAD samples. **(C)** Patients in TCGA were grouped by the median of C-Neural scores, then performed survival analysis based on PFI, DSS, and DFI data. *P* values were calculated by one-sided Wilcoxon rank-sum test in **(A)** and **(B)** and log-rank test in **(C)**, $P < 0.05$ was considered statistically significant.

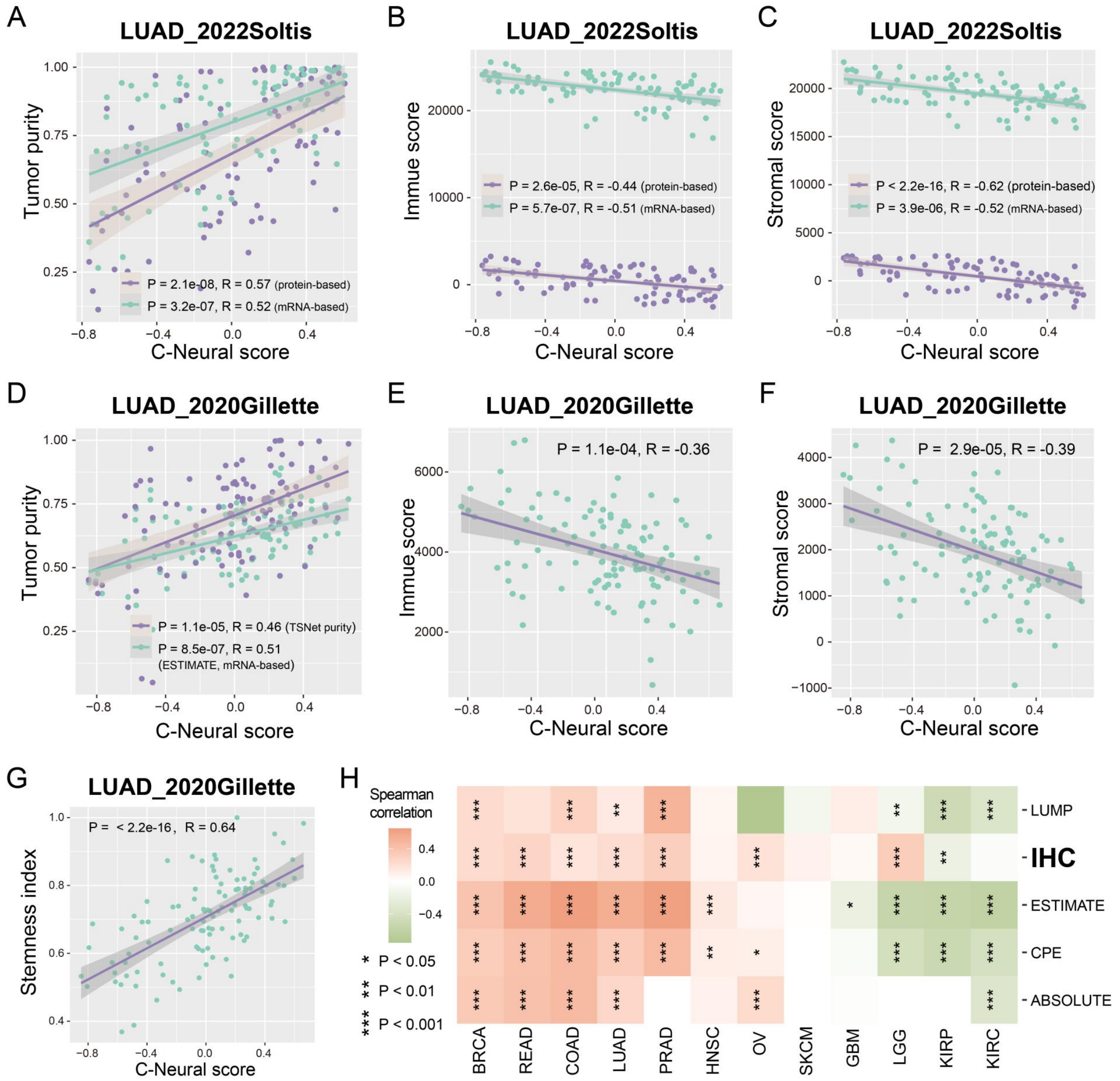

Figure S5. Correlation of C-Neural score with tumor microenvironment components.

(A)-(C) Correlation between C-Neural scores and tumor purity, immune scores, and stromal scores evaluated based on protein or gene expression in LUAD_2020Gillette dataset. **(D)-(G)** Correlation between C-Neural scores and tumor purity, immune scores, stromal scores and stemness index in LUAD_2020Gillette dataset. **(H)** Correlation between C-Neural scores and five tumor purity score of 13 cancer types in TCGA. *P* values were computed by Spearman's rank correlation analysis in **(A)-(H)**. *P* < 0.05 was considered statistically significant.

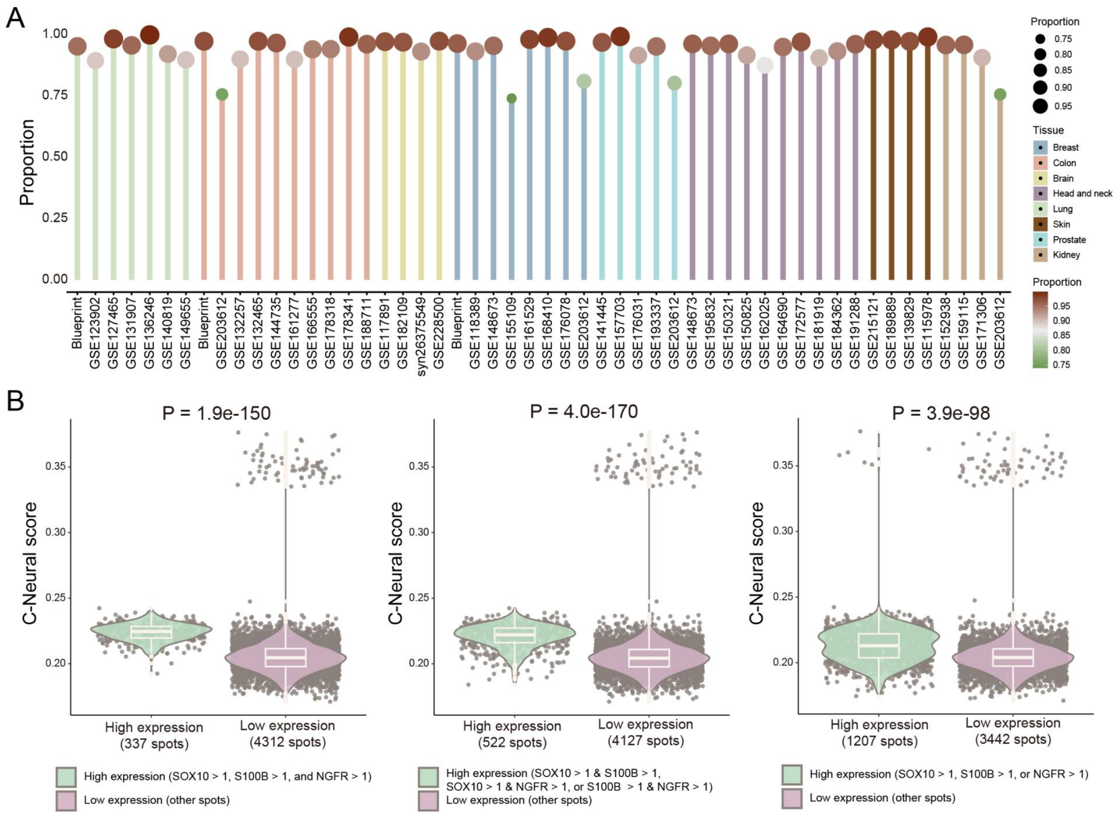

Figure S6. Proportion of expressed DEG-Ns and distribution of C-Neural scores in spatial transcriptomics.

(A) Proportion of DEG-Ns that were pass through quality control in pan-cancer scRNA-seq datasets. **(B)** Distribution of C-Neural scores between the regions that PNI and Schwann cells markers were high expression and other regions. *P* values were calculated by one-sided Wilcoxon rank-sum test in **(B)**, $P < 0.05$ was considered statistically significant.

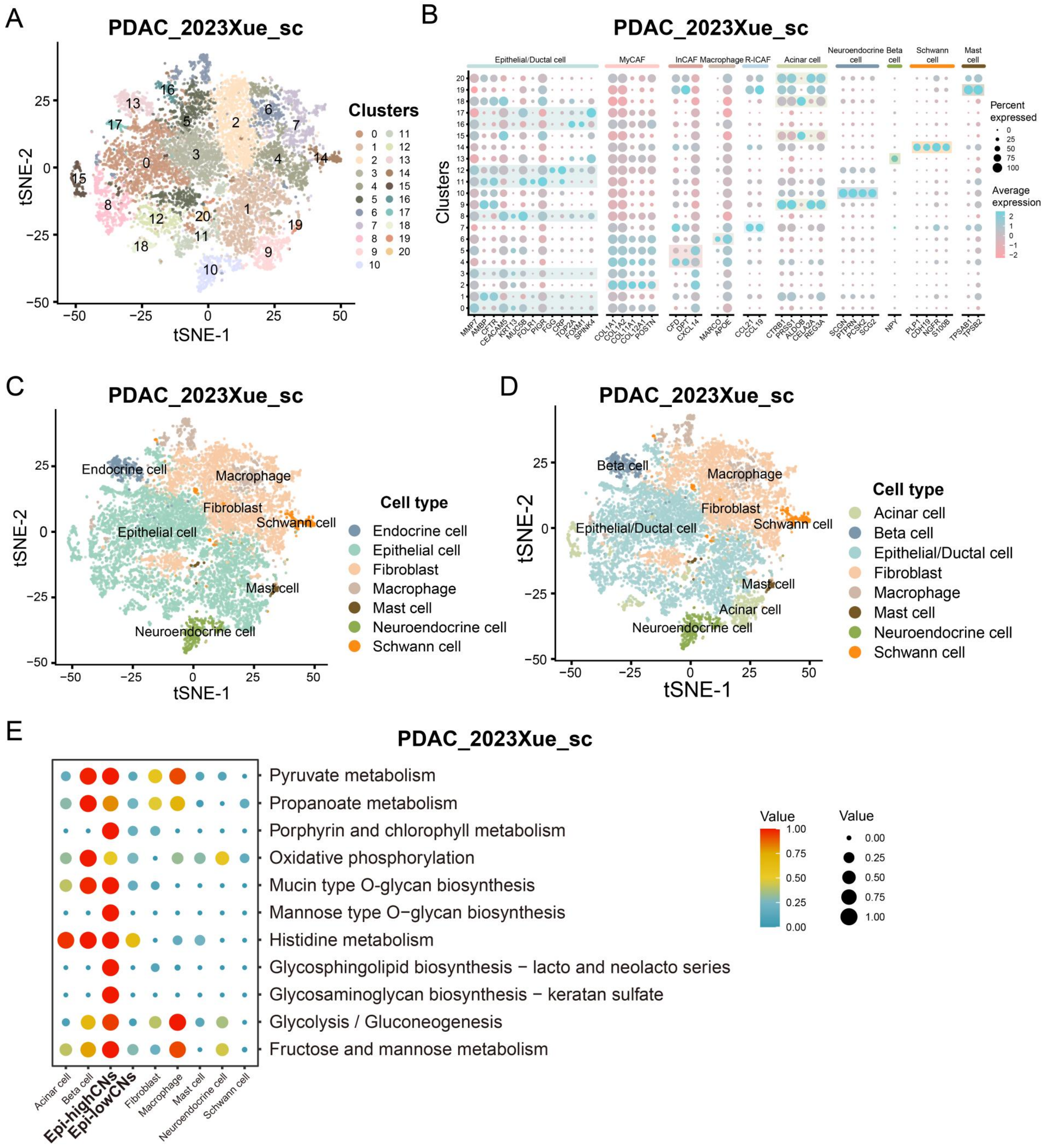

Figure S7. Cell annotation in PDAC_2023Xue_sc dataset.

(A) A total of 21 clusters identified in PDAC_2023Xue_sc dataset. **(B)** Expression of marker genes of cell types in clusters. **(C)** Annotation of cell types in PDAC_2023Xue_sc dataset. **(D)** Annotation of cell types, where "Epithelial/Ductal cells" were grouped into epi-highCNs and epi-lowCNs. **(E)** Differential metabolic pathway scores between epi-highCNs and epi-lowCNs.

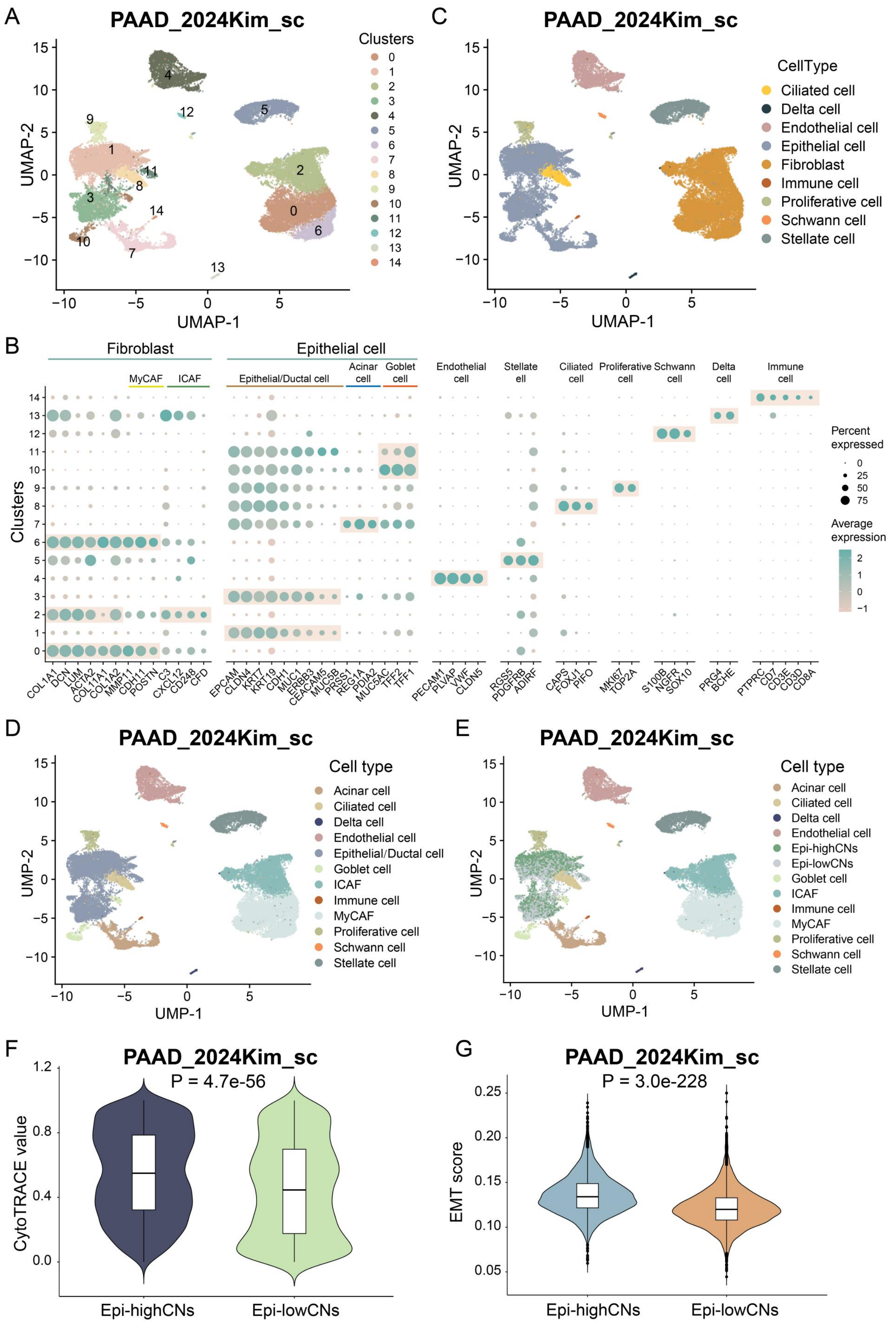

Figure S8. Cell annotation in PAAD_2024Kim_sc dataset.

(A) A total of 15 clusters identified in PAAD_2024Kim_sc dataset. **(B)** Expression of

marker genes of cell types in clusters. **(C)** Annotation of cell types. **(D)** Annotation of cell types, where major cell types were grouped into cell subtypes. **(E)** Annotation of cell types, where “Epithelial/Ductal cells” were grouped into epi-highCNs and epi-lowCNs. **(F)** Distribution of cell differentiation values by CytoTRACE method between epi-highCNs and epi-lowCNs. **(G)** Difference of EMT scores between epi-highCNs and epi-lowCNs. P values were calculated by one-sided Wilcoxon rank-sum test in **(F)** and **(G)**, $P < 0.05$ was considered statistically significant.

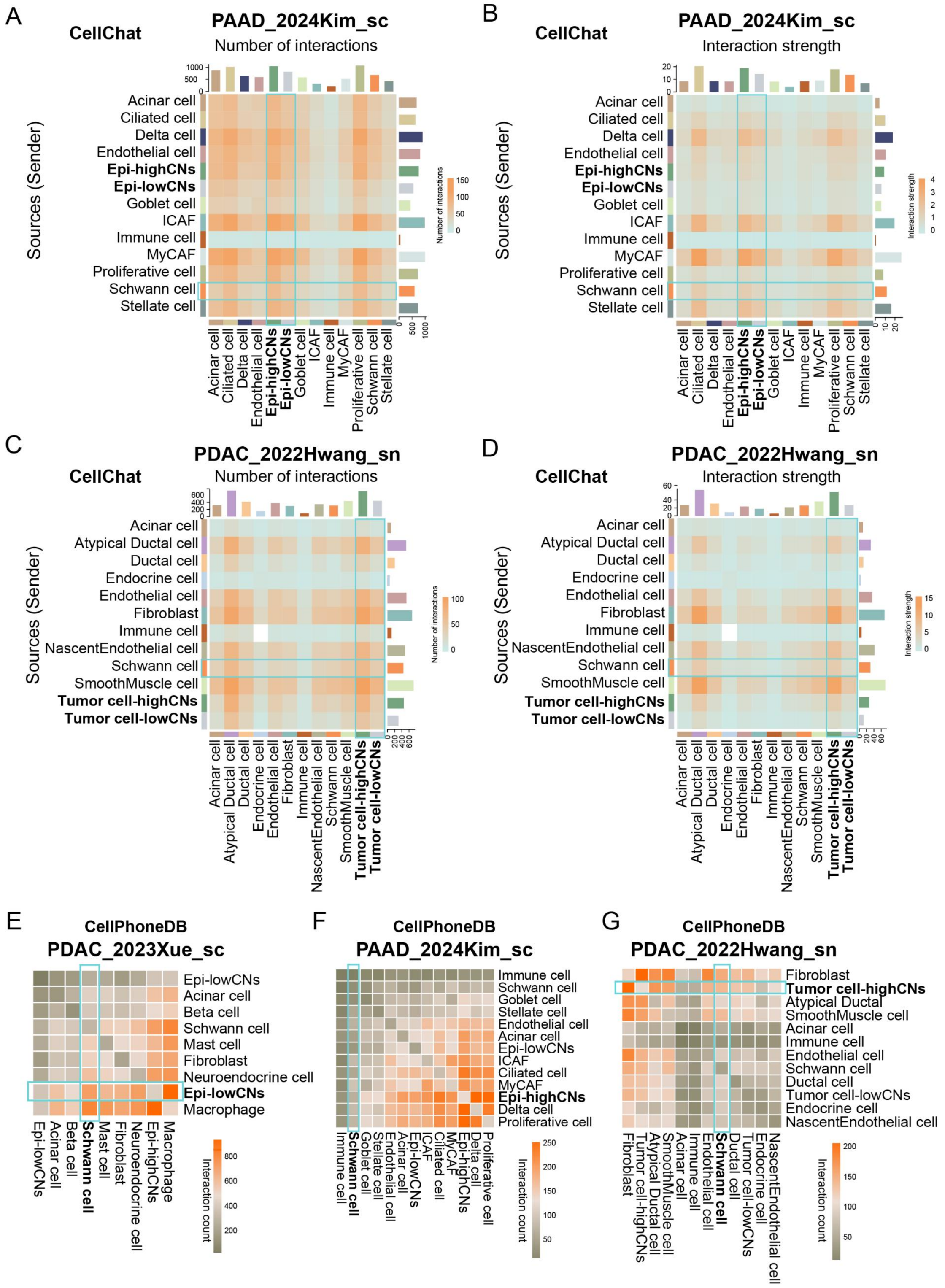

Figure S9. Cell-cell communications among cell types in PAAD scRNA-seq and snRNA-seq datasets.

(A) and **(B)** Cell-cell communications among cell types in PAAD_2024Kim_sc dataset, visualized in heatmaps where color indicates the number or strength of

communications. **(C)** and **(D)** Cell-cell communications among cell types in PDAC_2022Hwang_sn dataset, visualized in heatmaps where color indicates the number or strength of communications. **(E)-(G)** Cell-cell communications computed by CellPhoneDB method in PDAC_2023Xue_sc, PAAD_2024Kim_sc, and PDAC_2022Hwang_sn datasets.

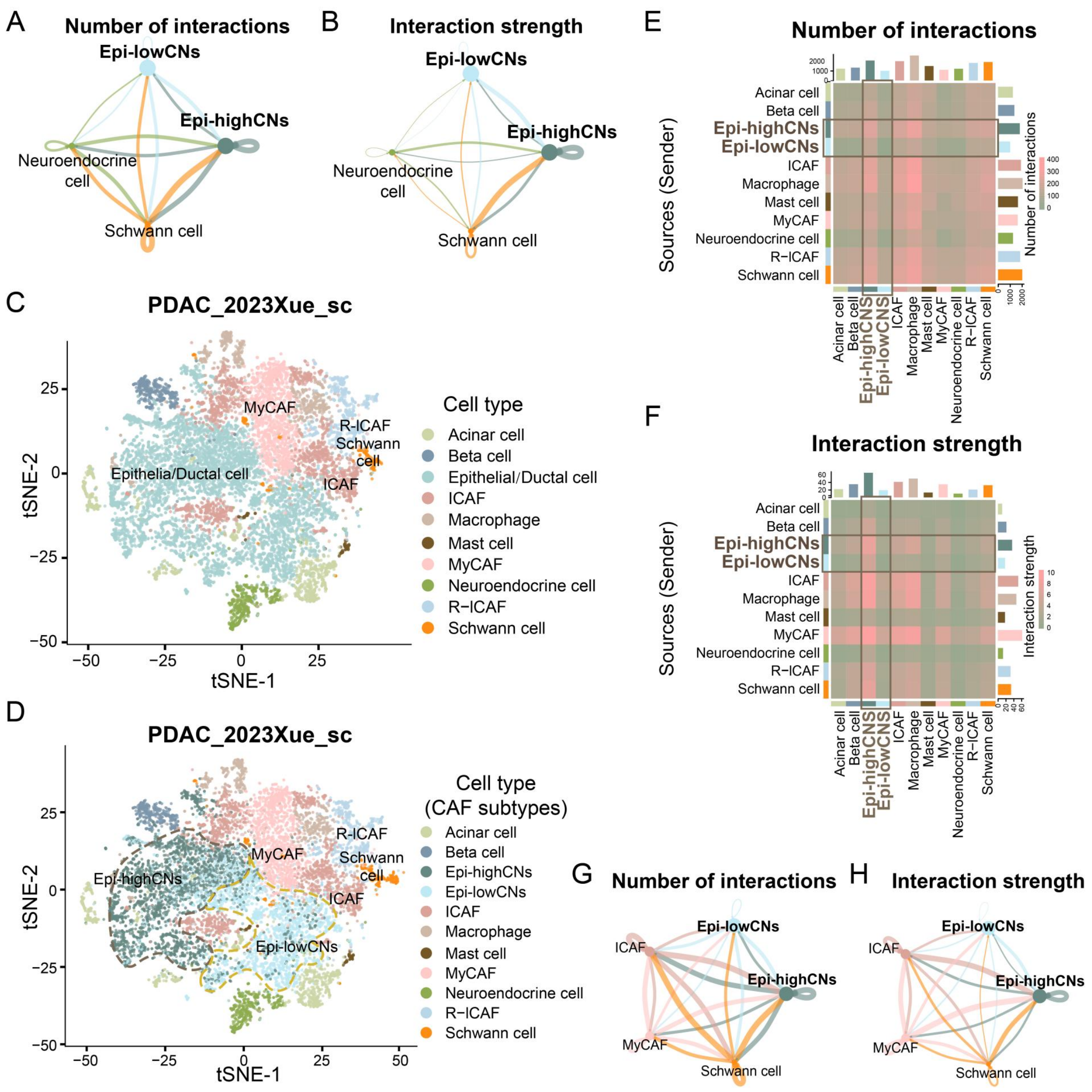

Figure S10. Cell-cell communications among cell types in PAAD_2024Kim_sc dataset.

(A) and **(B)** Cell-cell communications among epi-highCNs, epi-lowCNs, neuroendocrine cells, and Schwann cells, depicted by circle plot edge widths reflecting communication strength. **(C)** Annotation of cell types, where the CAFs were grouped into iCAFs, myCAFs, and r-lCAFs. **(D)** Annotation of cell types, where "Epithelial/Ductal cells" were grouped into epi-highCNs and epi-lowCNs. **(E)** and **(F)** Cell-cell communications among cell types. The color of heatmap indicates the number or strength of cell communications. **(G)** and **(H)** Cell-cell communications among epi-highCNs, epi-lowCNs, and CAF subtypes, shown in circle plots where edge widths denote communication strength.

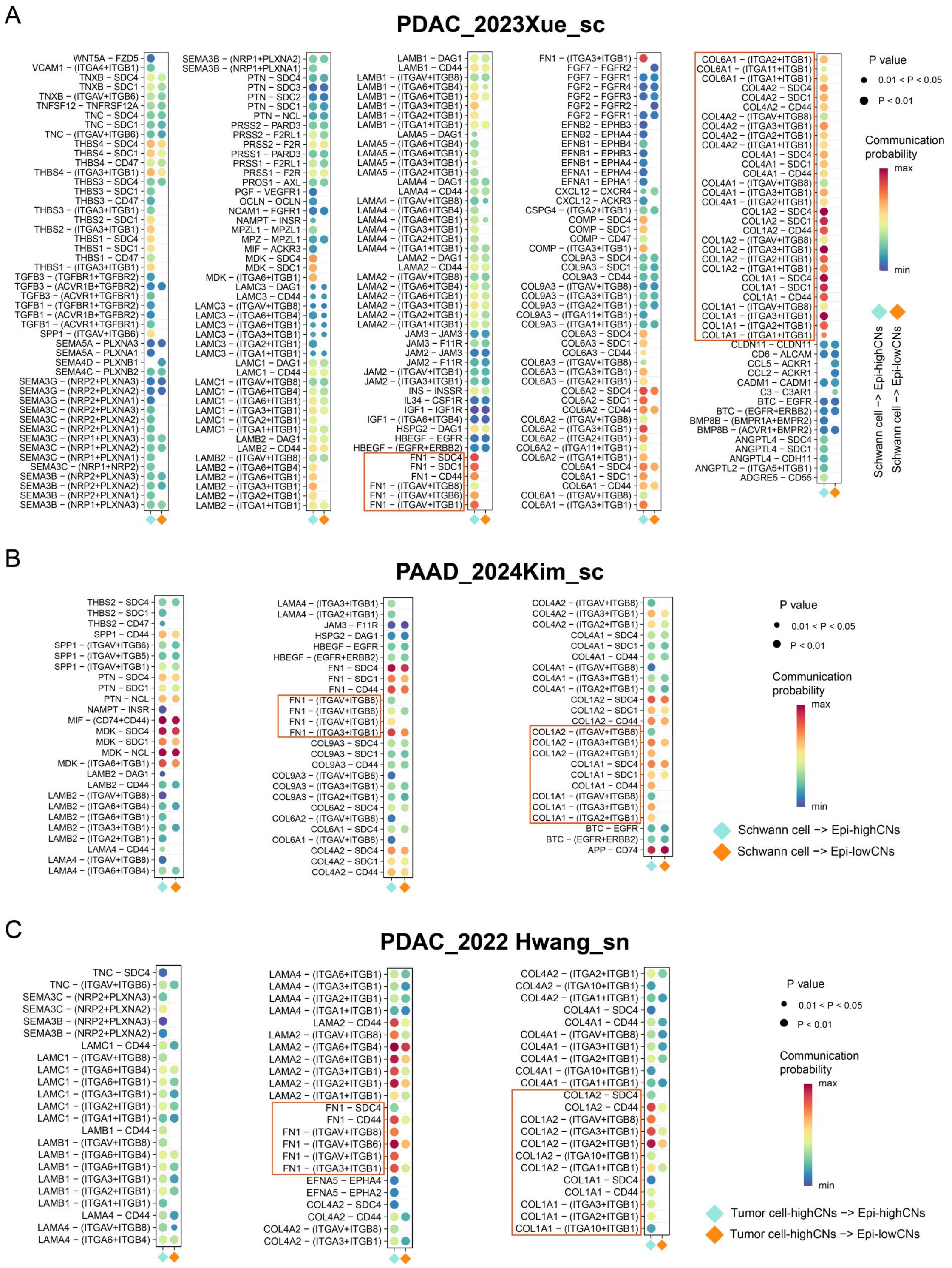

Figure S11. Significant ligand-receptor interactions in PAAD scRNA-seq and snRNA-seq datasets.

(A) and **(B)** Significant ligand-receptor interactions from Schwann cells to epi-highCNs and epi-lowCNs in PDAC_2023Xue_sc and PAAD_2024Kim_sc datasets. **(C)** Significant ligand-receptor interactions from Schwann cells to tumor

cell-highCNs and tumor cell-lowCNs in PDAC_2022 Hwang_sn dataset. *P* values were calculated using one-sided permutation test in (A)-(C). *P* < 0.05 was considered statistically significant.

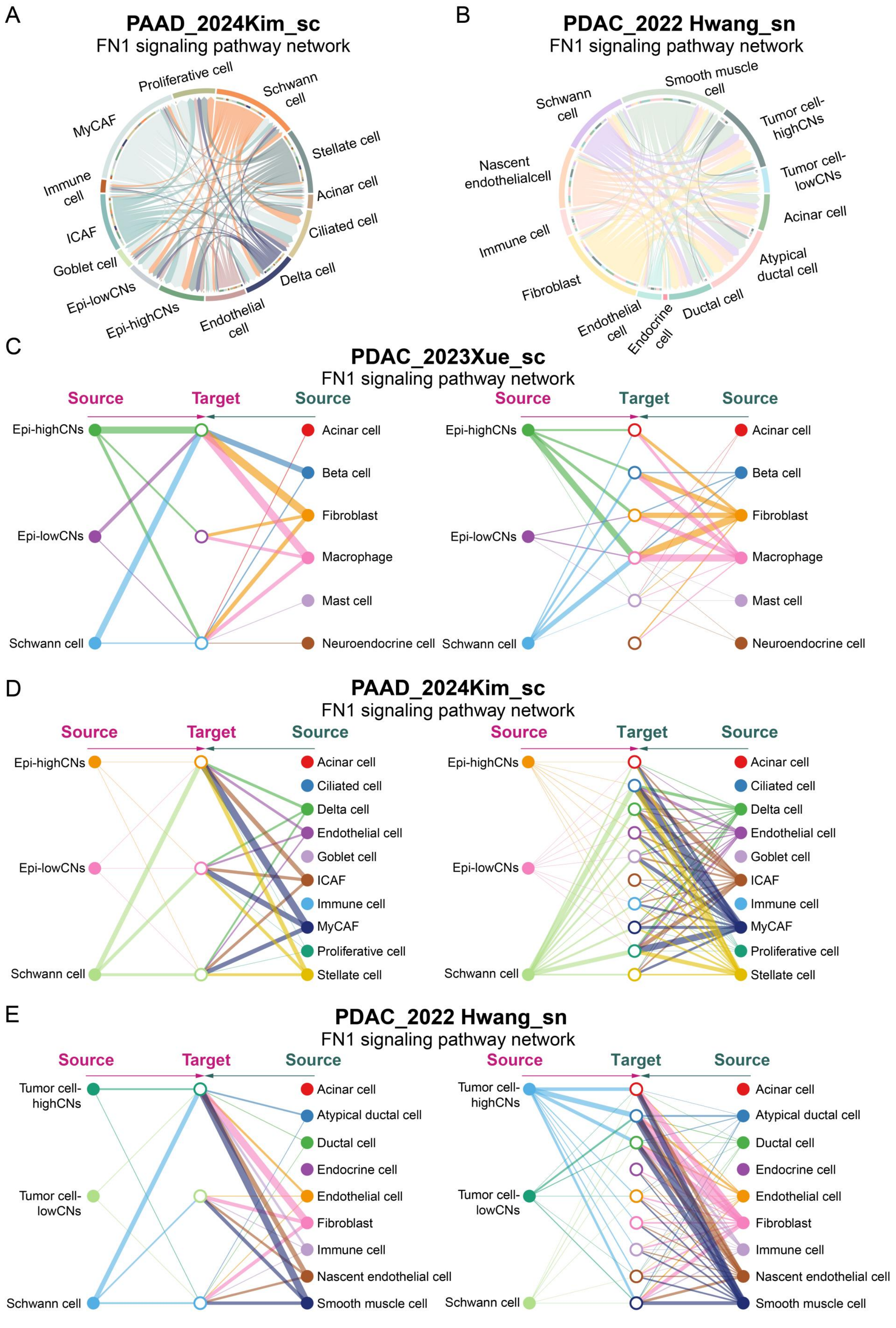

Figure S12. Cell-cell communications of FN1 signaling pathway among cell types in PAAD scRNA-seq and snRNA-seq datasets.

(A) and **(B)** Cell-cell communications of FN1 signaling pathway among all cell types in PAAD_2024Kim_sc and PDAC_2022 Hwang_sn datasets. **(C)** and **(D)** Cell-cell communications of FN1 signaling pathway from Schwann cell to epi-highCNs and epi-lowCNs, and among other cells in PDAC_2023Xue_sc and PAAD_2024Kim_sc datasets. **(E)** Cell-cell communications of FN1 signaling pathway from Schwann cell to tumor cell-highCNs and tumor cell-lowCNs, and among other cells in PDAC_2022 Hwang_sn dataset.

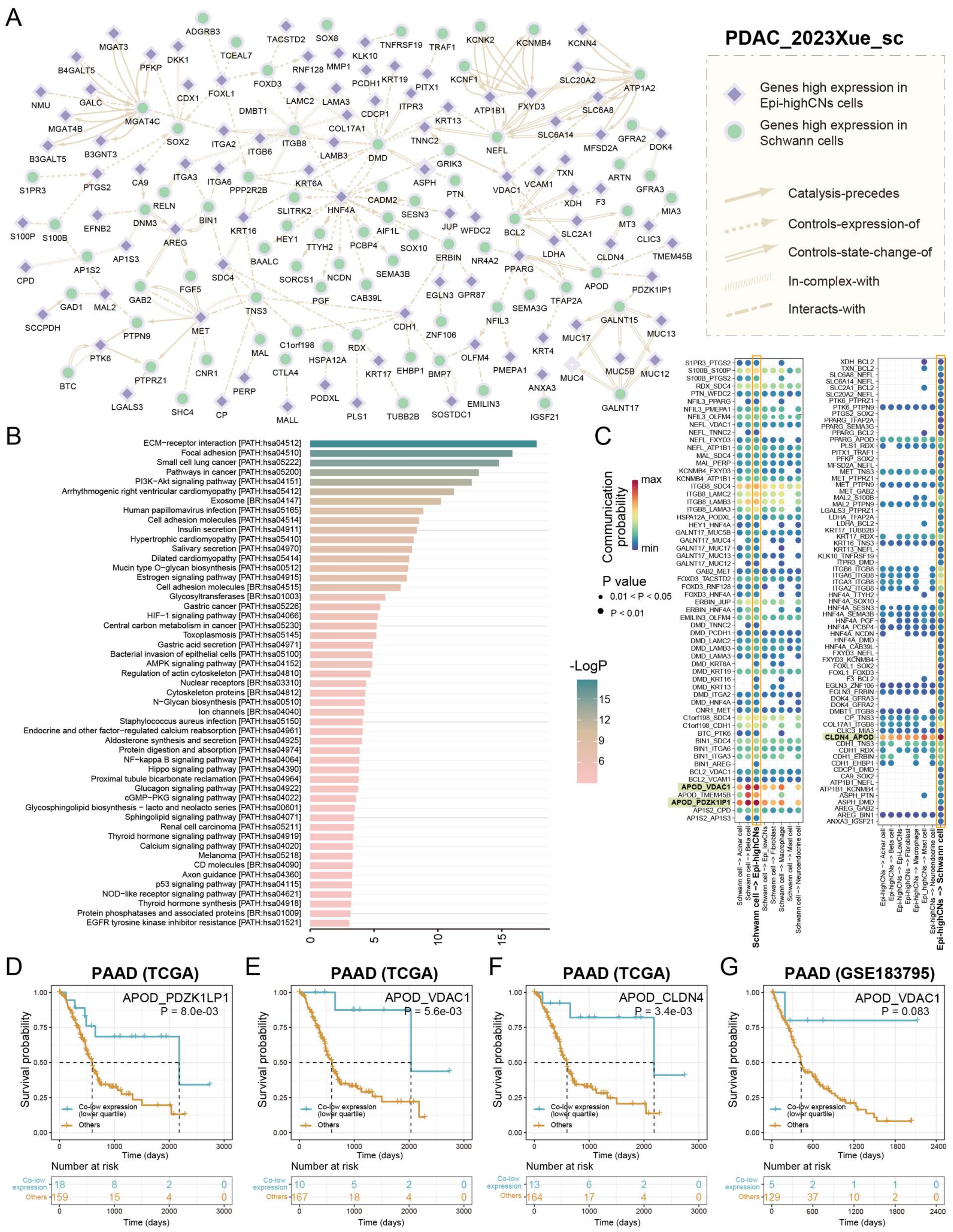

Figure S13. Protein-protein interactions between Schwann cells and epi-highCNs related to poor prognosis.

(A) The PPI network formed by marker genes of Schwann cells and epi-highCNs in PDAC_2023Xue_sc dataset. The Node type means marker genes derived from Schwann cells or epi-highCNs. **(B)** The KEGG functional enrichment analysis for

genes in PPI network. **(C)** The communication probability of PPI between Schwann cells and epi-highCNs inferred by CellChat method. The color of dots means communication probability. **(D)** and **(F)** Samples were grouped into gene pair (*APOD_PDZK1LP1*, *APOD_VDAC1*, or *APOD_CLDN4*) co-low expression and others in PAAD_TCGA, log-rank test was used to evaluate the difference of prognosis. **(G)** Samples were grouped into gene pair *APOD_VDAC1* co-low expression and others in PAAD_GSE183795, log-rank test was used to evaluate the difference of prognosis. *P* values were computed by hypergeometric test in **(B)**, one-sided permutation test in **(C)**, and log-rank test in **(D)**-**(G)**. $P < 0.05$ was considered statistically significant.

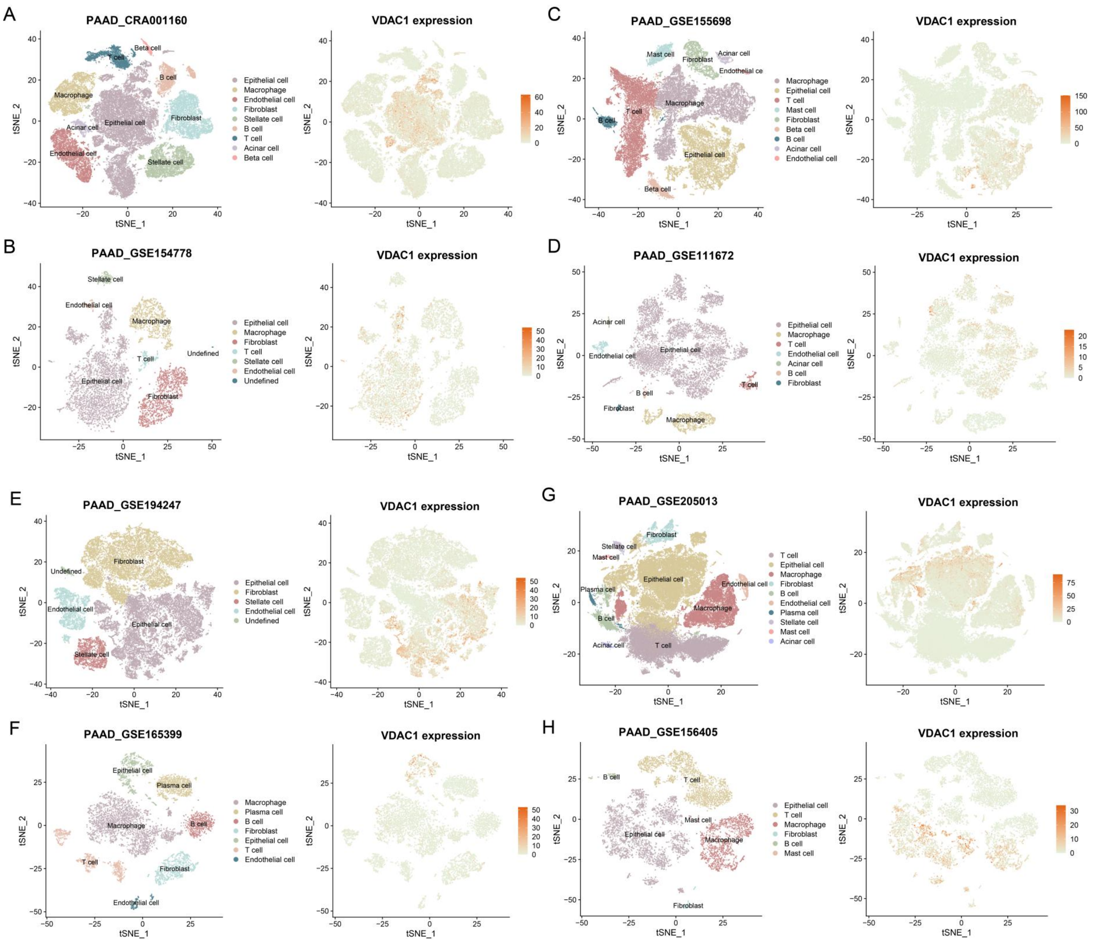

Figure S14. Expression of *VDAC1* among cell types in PAAD scRNA-seq datasets.

(A)-(H) Cell types and expression of VDAC1 among cell types in PAAD scRNA-seq datasets.

.

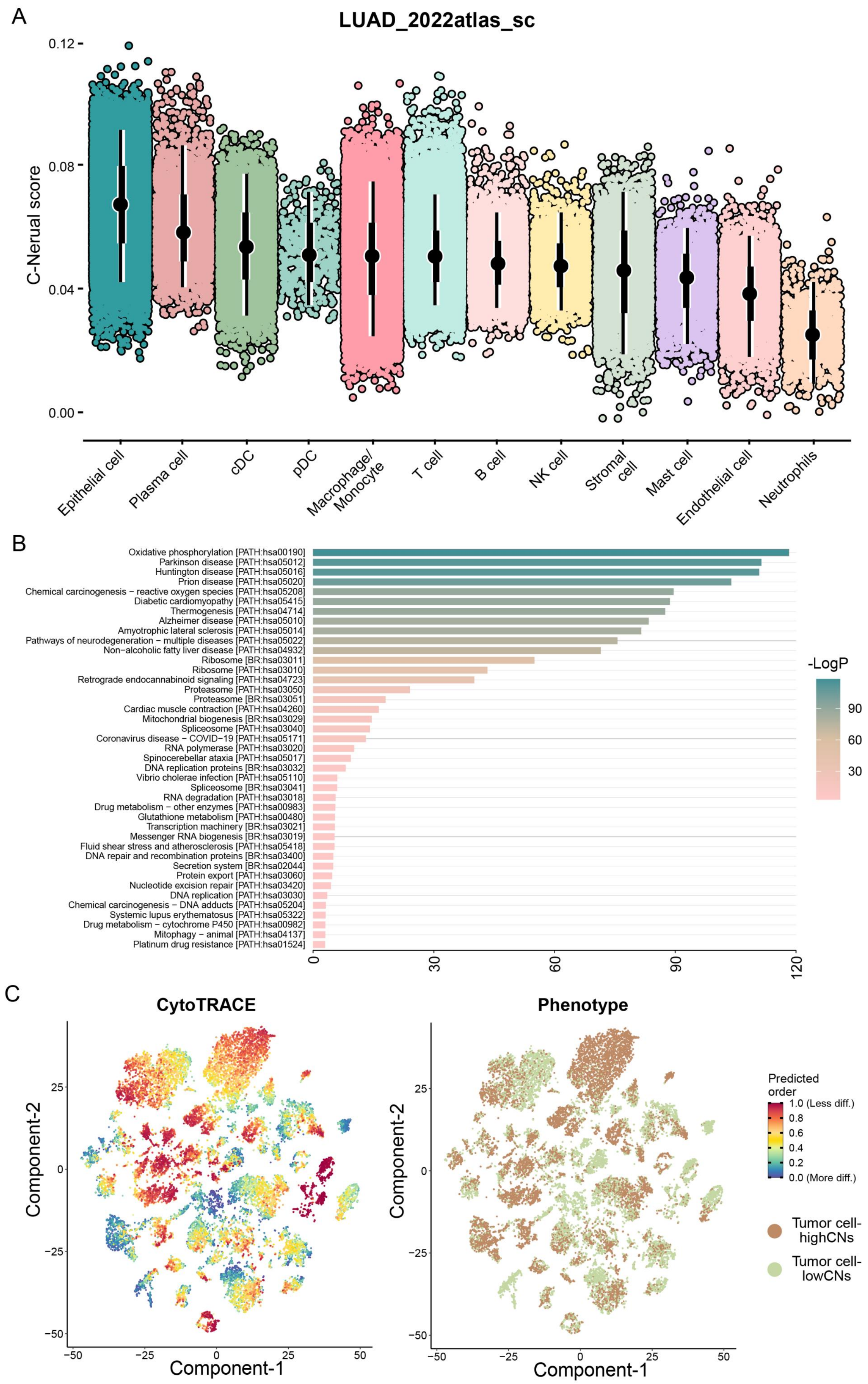
A
LUAD_2022atlas_sc
C-Nerual score
0.12
0.08
0.04
0.00
Epithelial cell
Plasma cell
cDC
pDC
Macrophage/ Monocyte
T cell
B cell
NK cell
Stromal cell
Mast cell
Endothelial cell
Neutrophils
B
Oxidative phosphorylation [PATH:hsa00190]
Parkinson disease [PATH:hsa05012]
Huntington disease [PATH:hsa05016]
Prion disease [PATH:hsa05020]
Chemical carcinogenesis − reactive oxygen species [PATH:hsa05208]
Diabetic cardiomyopathy [PATH:hsa05415]
Thermogenesis [PATH:hsa04714]
Alzheimer disease [PATH:hsa05010]
Amyotrophic lateral sclerosis [PATH:hsa05014]
Pathways of neurodegeneration − multiple diseases [PATH:hsa05022]
Non−alcoholic fatty liver disease [PATH:hsa04932]
Ribosome [BR:hsa03011]
Ribosome [PATH:hsa03010]
Retrograde endocannabinoid signaling [PATH:hsa04723]
Proteasome [PATH:hsa03050]
Proteasome [BR:hsa03051]
Cardiac muscle contraction [PATH:hsa04260]
Mitochondrial biogenesis [BR:hsa03029]
Spliceosome [PATH:hsa03040]
Coronavirus disease − COVID−19 [PATH:hsa05171]
RNA polymerase [PATH:hsa03020]
Spinocerebellar ataxia [PATH:hsa05017]
DNA replication proteins [BR:hsa03032]
Vibrio cholerae infection [PATH:hsa05110]
Spliceosome [BR:hsa03041]
RNA degradation [PATH:hsa03018]
Drug metabolism − other enzymes [PATH:hsa00983]
Glutathione metabolism [PATH:hsa00480]
Transcription machinery [BR:hsa03021]
Messenger RNA biogenesis [BR:hsa03019]
Fluid shear stress and atherosclerosis [PATH:hsa05418]
DNA repair and recombination proteins [BR:hsa03400]
Secretion system [BR:hsa02044]
Protein export [PATH:hsa03060]
Nucleotide excision repair [PATH:hsa03420]
DNA replication [PATH:hsa03030]
Chemical carcinogenesis − DNA adducts [PATH:hsa05204]
Systemic lupus erythematosus [PATH:hsa05322]
Drug metabolism − cytochrome P450 [PATH:hsa00982]
Mitophagy − animal [PATH:hsa04137]
Platinum drug resistance [PATH:hsa01524]
0
30
60
90
120
-LogP
90
60
30
C
CytoTRACE
Phenotype
Component-2
Component-1
25
0
−25
−50
50
Predicted order
1.0 (Less diff.)
0.8
0.6
0.4
0.2
0.0 (More diff.)
Tumor cell-highCNs
Tumor cell-lowCNs

Figure S15. Evaluation of neural signal in LUAD scRNA-seq dataset.

(A) Distribution of C-Neural scores among all cell types in LUAD_2022atlas_sc dataset. **(B)** Tumor cells were grouped into tumor cell-highCNs and tumor cell-lowCNs according to the median of C-Neural scores, KEGG functional enrichment analysis for top 300 DEGs. **(C)** Cell differentiation level evaluated by CytoTRACE method, and corresponding neural signal-related tumor cell subtypes. *P* values were computed by hypergeometric test in **(B)**. $P < 0.05$ was considered statistically significant.

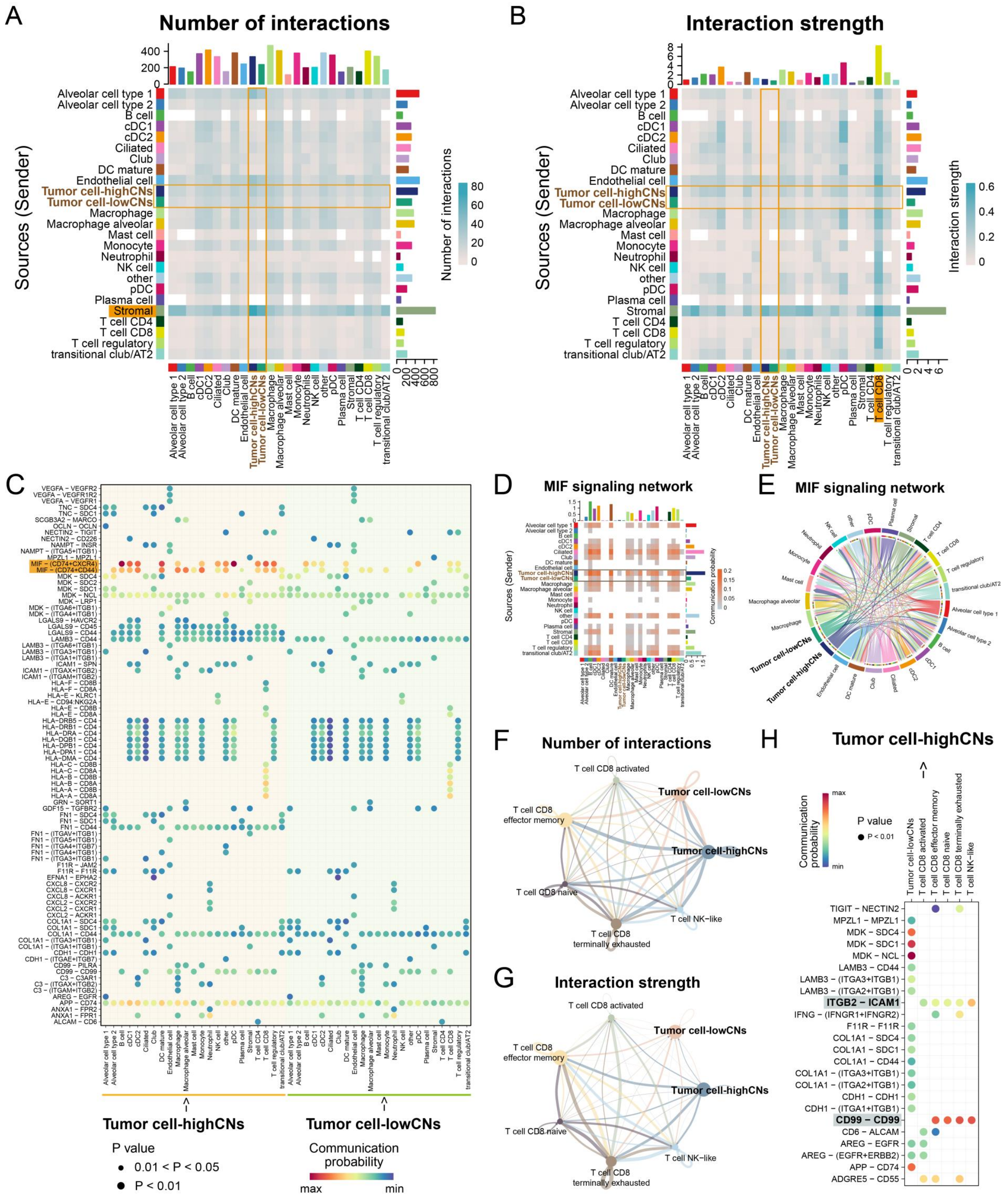

Figure S16. Analysis of cell-cell communications in LUAD scRNA-seq dataset.

(A) and **(B)** The cell-cell communications among cell types in LUAD_2022atlas_sc dataset. The color of heatmap indicates the number or strength of cell communications. **(C)** All significant ligand-receptor pairs contributing to the signaling sending tumor cell-highCNs or tumor cell-lowCNs to other cells. The dot color and size represent the calculated communication probability and *P* value. **(D)** The inferred MIF signaling network. The color of heatmap represents the communication

probability. **(E)** The chord diagram shows the communications of the MIF signaling pathway inferred by aggregating all ligand-receptor pairs among all cell subtypes. **(F)** and **(G)** Cell-cell communications among tumor cell-highCNs, tumor cell-lowCNs, and immune cells in LUAD_2022atlas_sc dataset. The width of edge in circle plot indicates the number or strength of cell-cell communications. **(H)** The significant ligand-receptor pairs that contribute to the signaling sending immune cells to tumor cell-highCNs. The dot color and size represent the calculated communication probability and *P* values. *P* values are computed from one-sided permutation test in **(C)** and **(H)**. $P < 0.05$ was considered statistically significant.

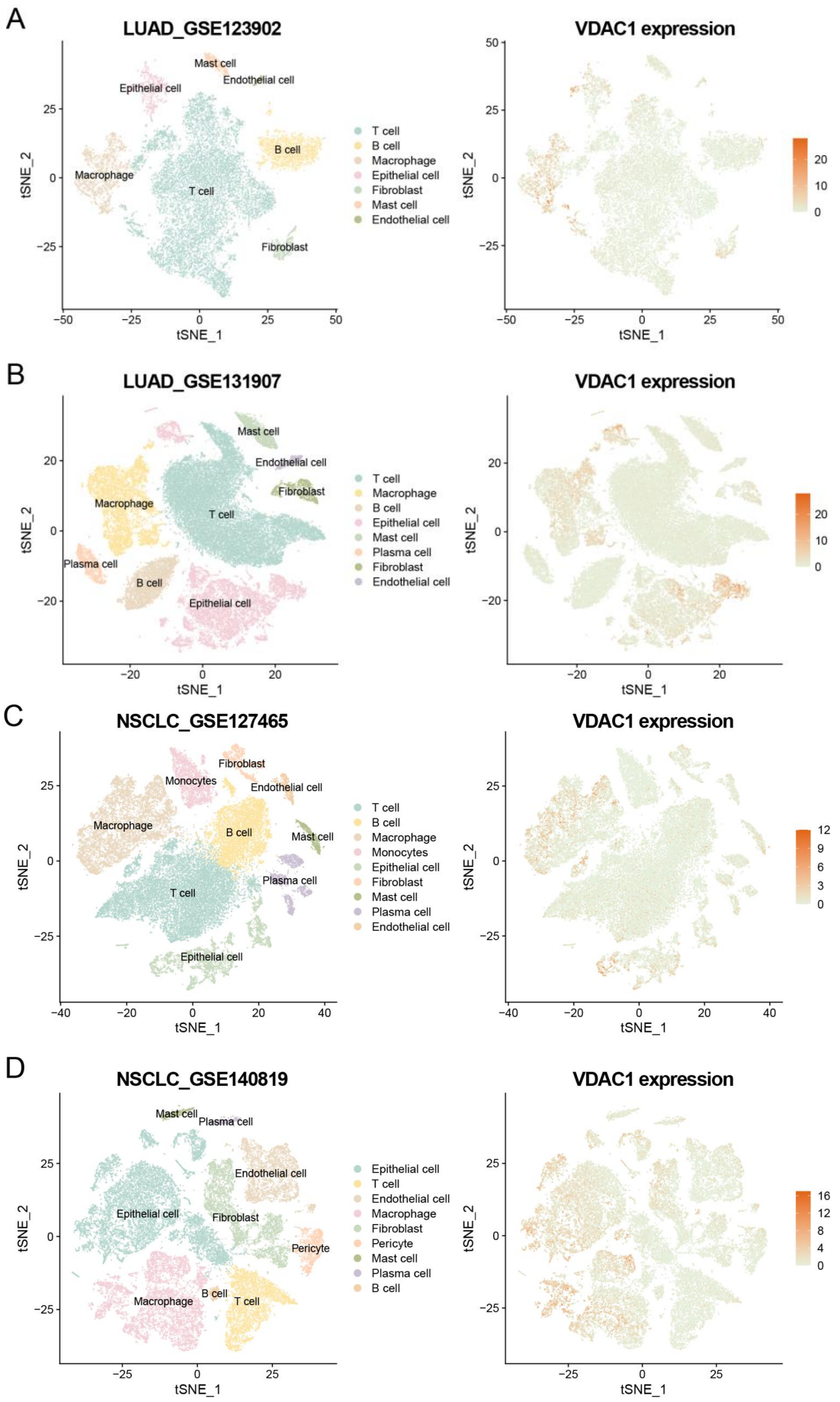

Figure S17. Expression of VDAC1 among cell types in LUAD and NSCLC scRNA-seq datasets.

(A)-(D) Cell types and expression of VDAC1 among cell types in LUAD and NSCLC scRNA-seq datasets。

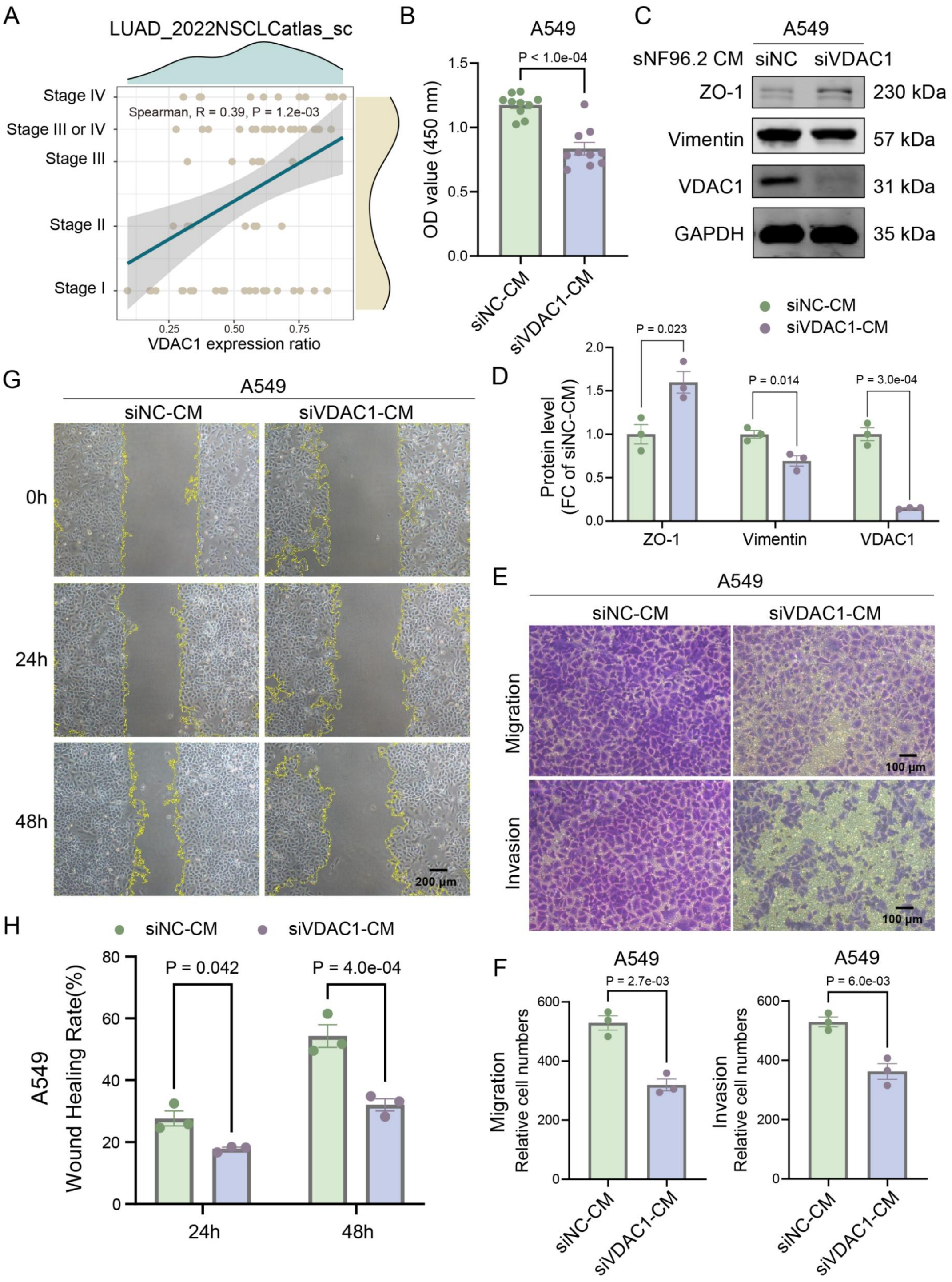

Figure S18. Schwann cells promote LUAD progression mediate by VDAC1.

(A) Proportion of VDAC1 expression in epithelial cell is positively correlated with LUAD stage in LUAD_2022NSCLCatlas_sc dataset. **(B)** The CCK8 assay examines the proliferation of A549-siVDAC1 and A549-siNC in SC-CM (n=10). **(C)** and **(D)** Western blot analysis for A549-siVDAC1 and A549-siNC in SC-CM (n=3). **(E)** and

(F) Transwell assays for detection of migration and invasion in A549-siVDAC1 and A549-siNC in SC-CM (n=3). **(G)** and **(H)** The scratch assays for A549-siVDAC1 and A549-siNC in SC-CM (n=3). *P* values were calculated by Student's t test in **(B)**, **(D)**, **(F)**, and **(H)**, $P < 0.05$ was considered statistically significant.

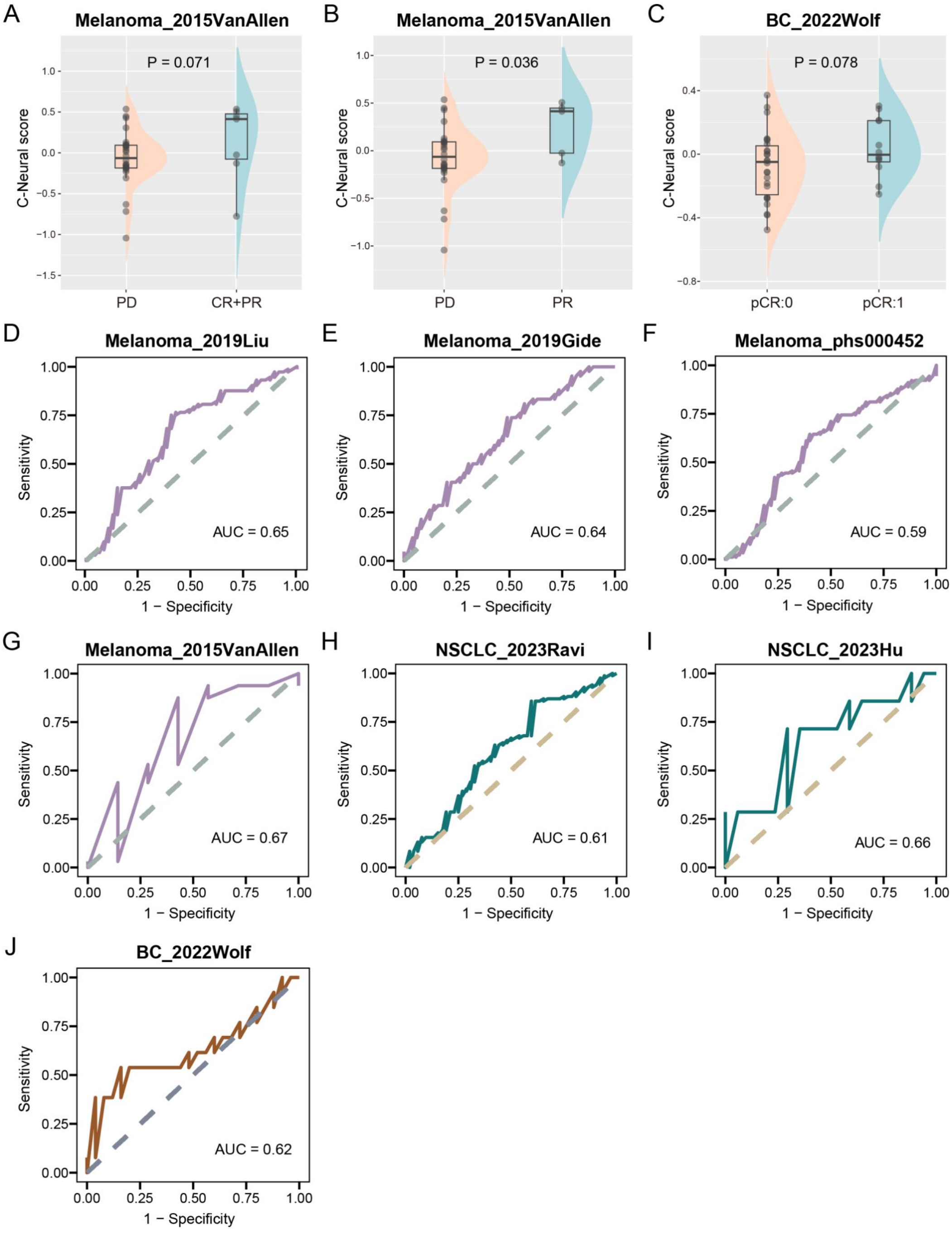

Figure S19. Prediction of immunotherapy response by C-Neural score.

(A) and **(B)** Distribution of C-Neural scores between responsive and non-responsive patients in Melanoma_2015VanAllen dataset. **(C)** Distribution of C-Neural scores

between responsive and non-responsive basal breast cancer patients in BC_2022Wolf dataset. **(D)-(J)** The ROC curves for predicted performance of immunotherapy response by C-Neural scores in melanoma, NSCLC, and BC datasets.

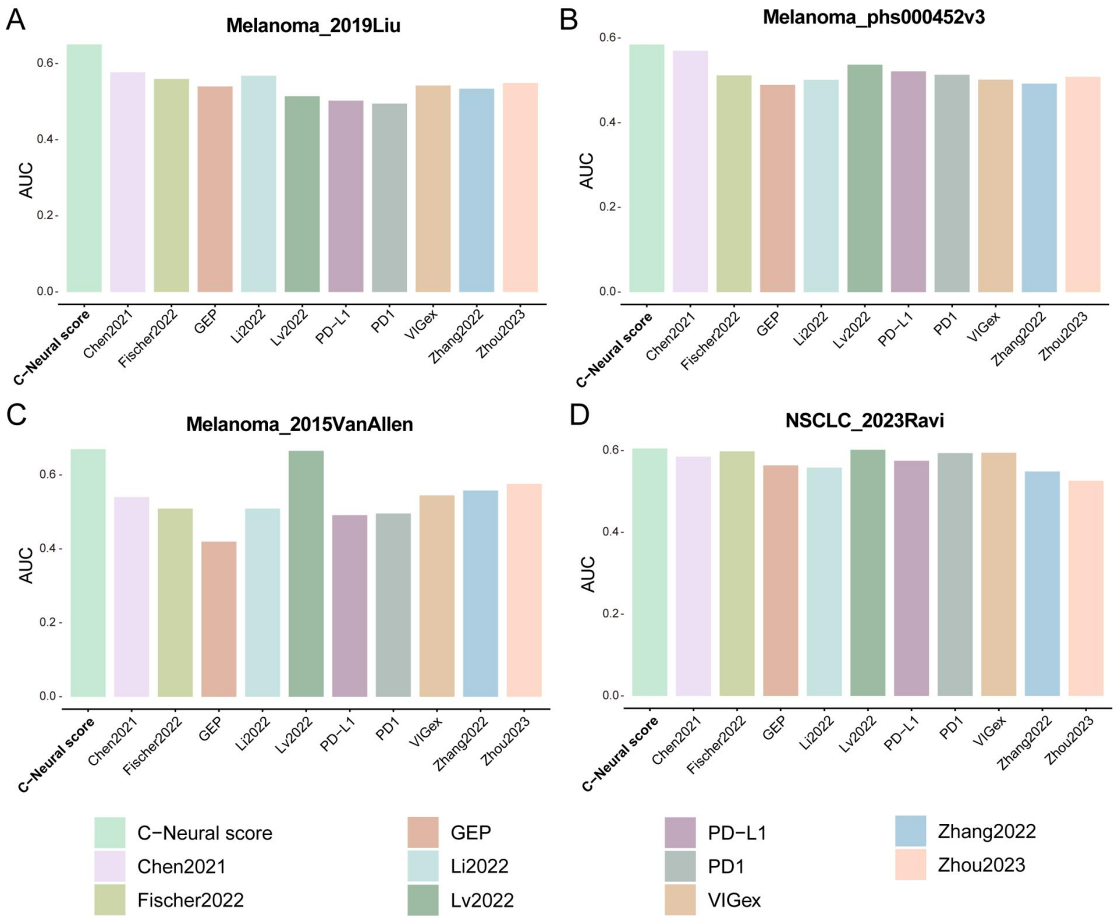

Figure S20. Performance of C-Neural scores compared with other transcriptomic signatures or algorithms.

(A)-(D) The AUC values of ROC curves of predicted algorithms for immunotherapy response in melanoma, NSCLC, and BC datasets.

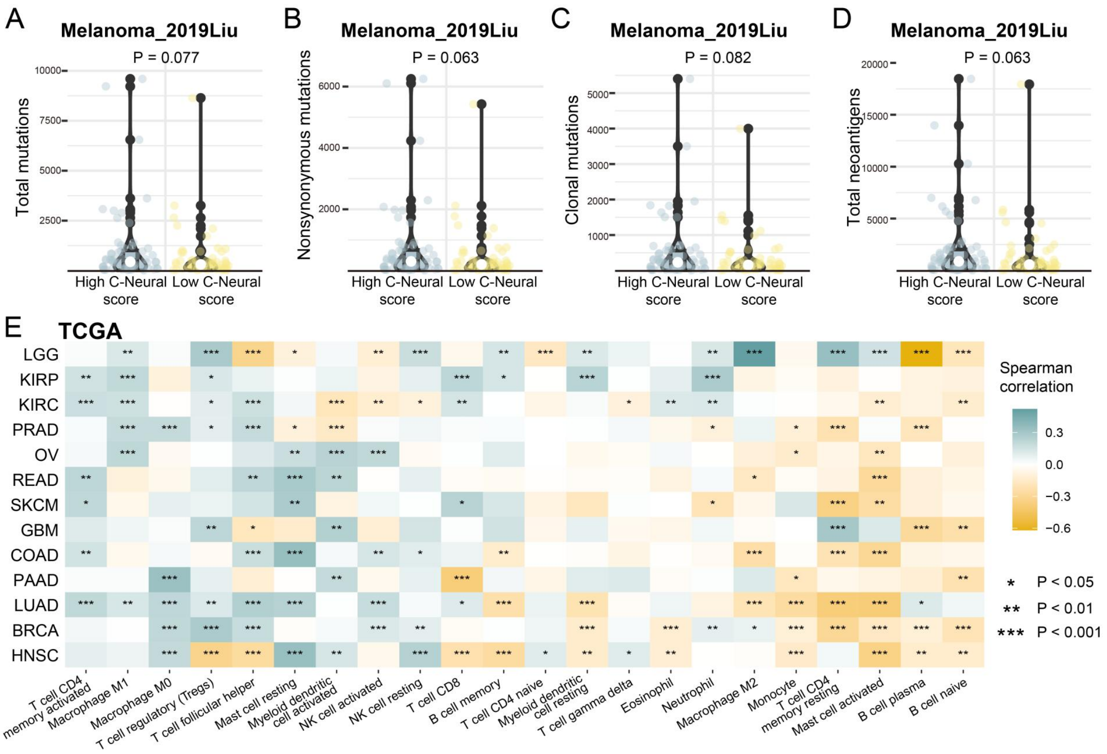

Figure S21. Correlation of C-Neural score and tumor mutational burden and immune infiltration.

(A)-(D) The violin plots show the difference of "Total mutations", "Nonsynonymous mutations", "Clonal mutations", and "Total neoantigens" between high and low C-Neural score groups in Melanoma_2019Liu dataset. **(E)** Correlation between C-Neural scores and infiltration of immune cells in TCGA dataset. *P* values were calculated by one-sided Wilcoxon rank-sum test in **(A)-(D)** and Spearman's rank correlation analysis in **(E)**. $P < 0.05$ was considered statistically significant.

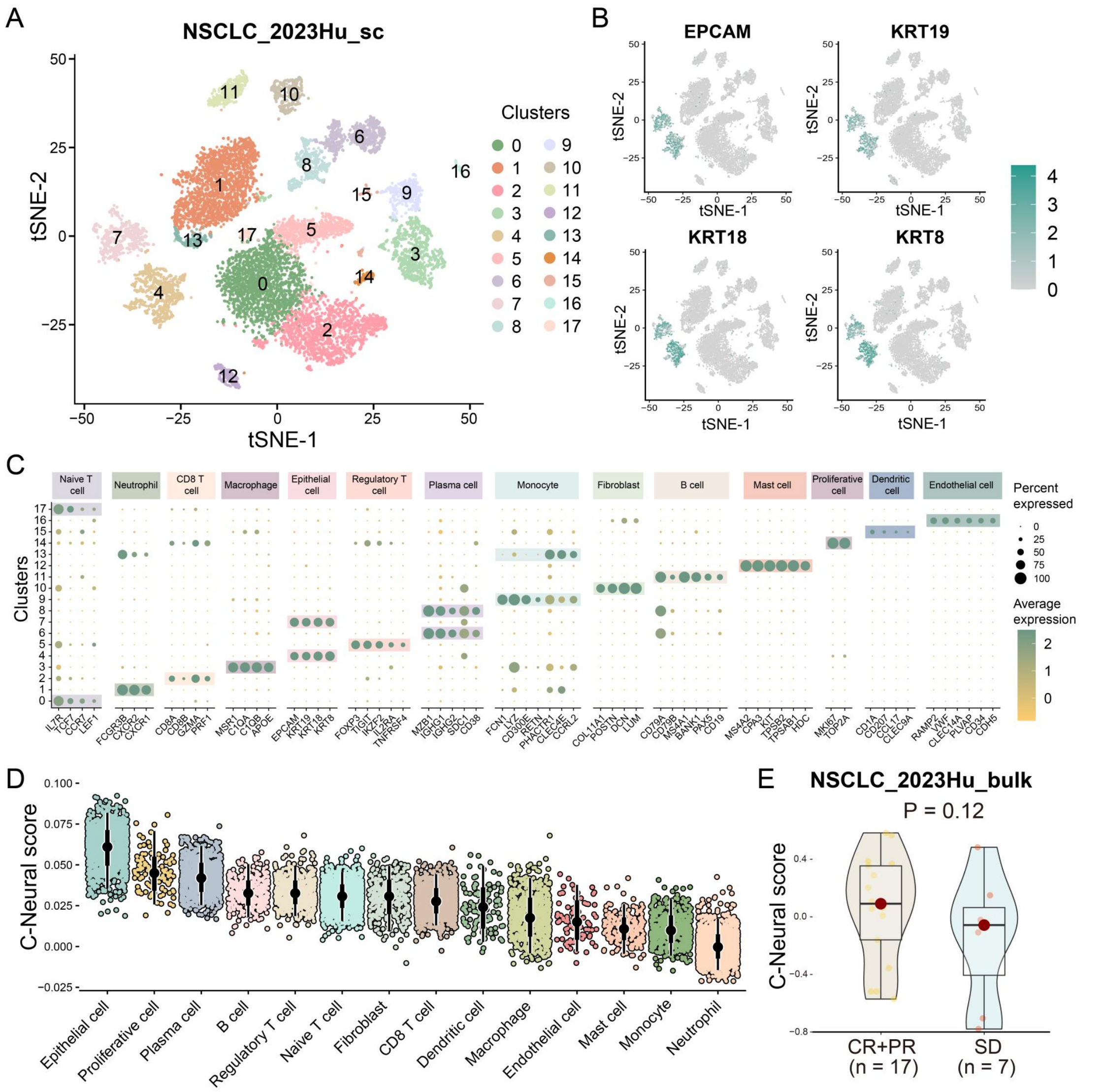

Figure S22. Cell type annotation in NSCLC immunotherapy scRNA-seq data.

(A) A total of 18 clusters were identified in NSCLC immunotherapy scRNA-seq data. **(B)** Distribution of epithelial cell markers (*EPCAM*, *KRT18*, *KRT19*, and *KRT8*) among clusters. **(C)** Clusters identified and annotated into 14 cell types for three immunotherapy pre-treatment samples in NSCLC_2023Hu_sc dataset. **(D)** Distribution of C-Neural score among cell types. **(E)** The difference in C-Neural score between responsive (CR and PR) patents and non-responsive (SD) patients based on immunotherapy NSCLC_2023Hu_bulk dataset. *P* values were calculated by one-sided Wilcoxon rank-sum test in **(E)**, $P < 0.05$ was considered statistically significant.

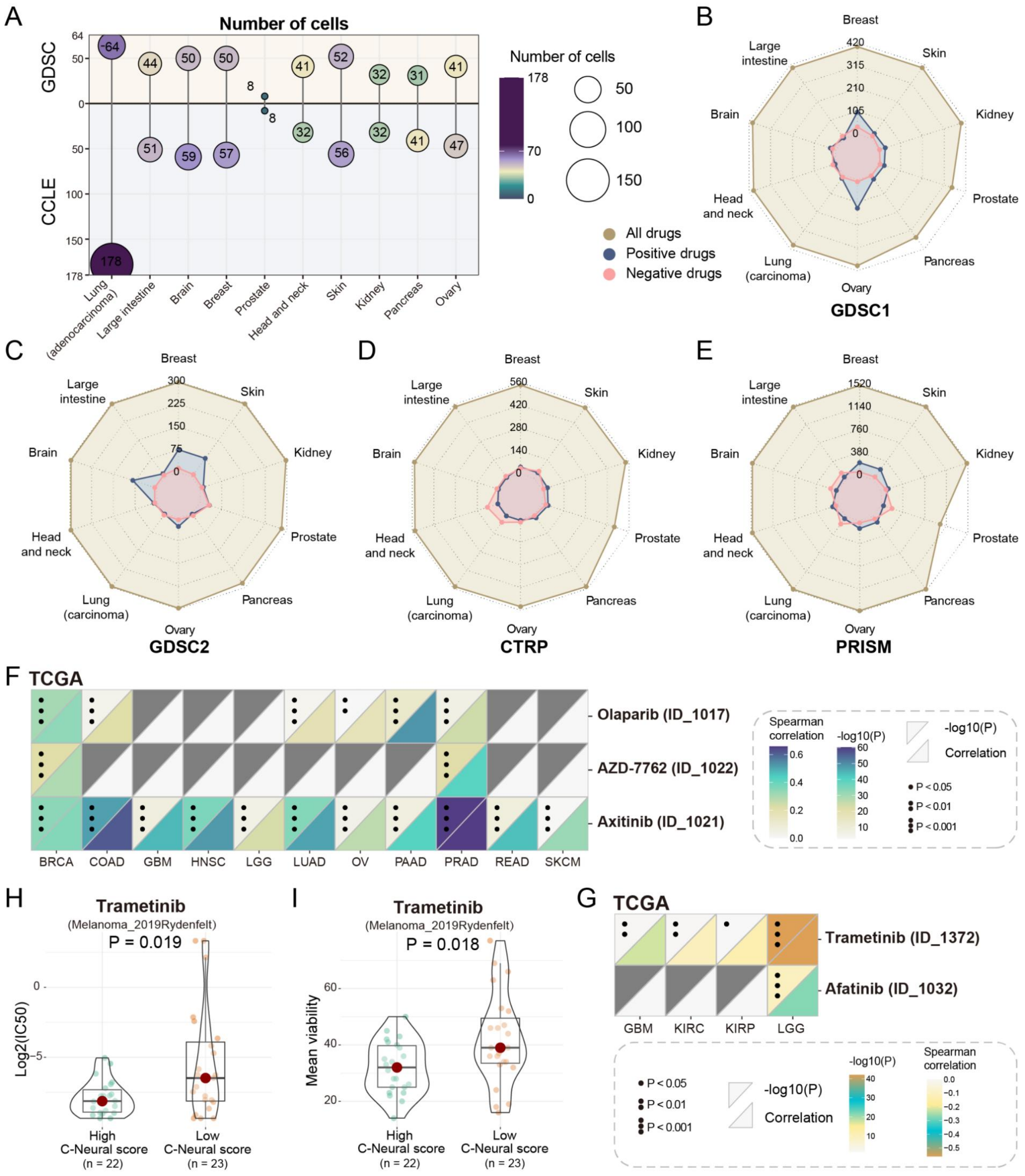

Figure S23. Identification of drugs for cancer based on pharmacological screening datasets.

(A) Number of cancer cell lines among ten tissues in GDSC and CCLE data resource. **(B)-(E)** The number of resistant or sensitive drugs which were correlation with C-Neural score in GDSC1, GDSC2, CTRP, and PRISM Repurposing datasets. **(F)** The oncoPredict values of Olaparib, AZD-7762, and Axitinib were significant positive correlation with C-Neural score in TCGA. **(G)** The oncoPredict values of Trametinib and Afatinib were significant negative correlation with C-Neural score in TCGA. **(H)**

The IC50 values of Trametinib between high and low C-Neural score samples. **(I)** The "Mean viability" values of cell lines treated by Trametinib between high and low C-Neural score samples. *P* values were calculated by Spearman's rank correlation analysis in **(F)** and **(G)**, one-sided Wilcoxon rank-sum test in **(H)** and **(I)**. $P < 0.05$ was considered statistically significant.

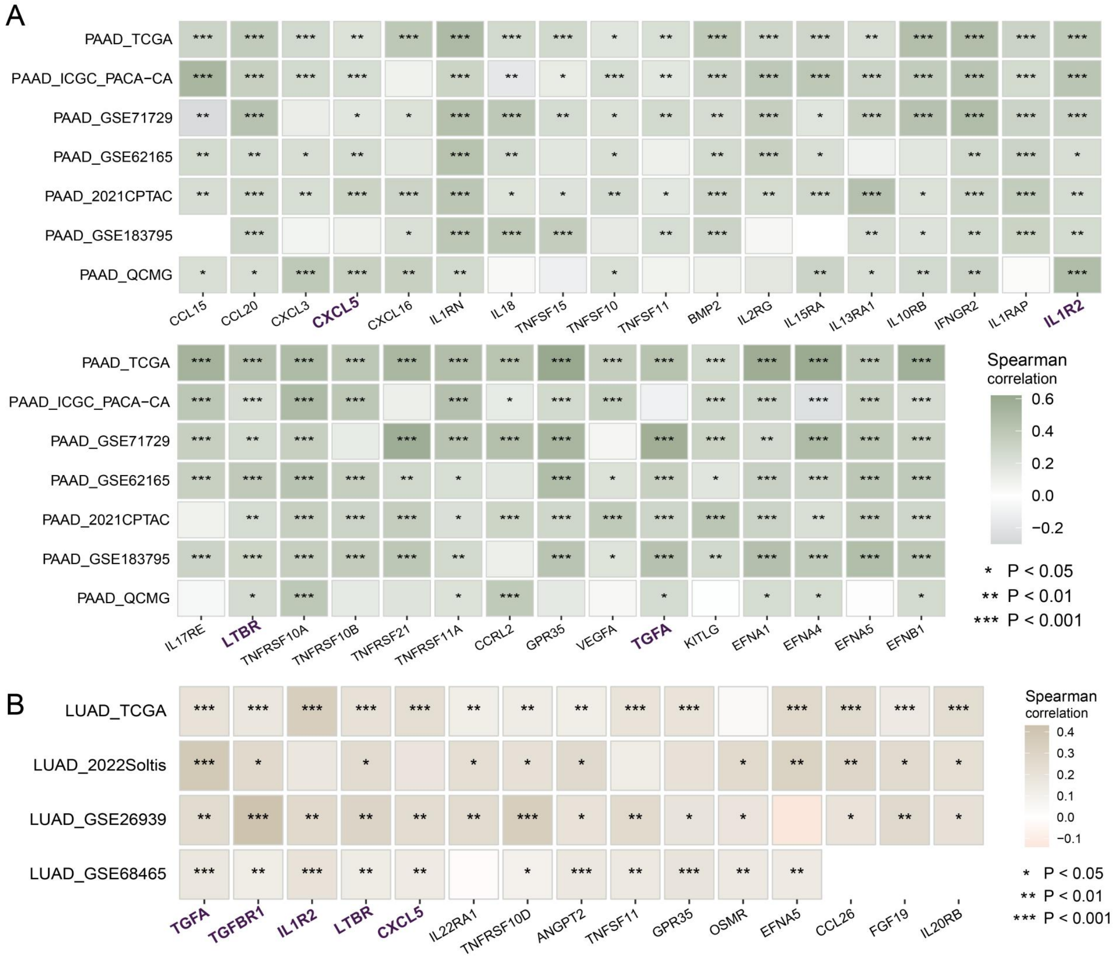

Figure S24. Correlation analysis for expression of *VDAC1* and cytokines.

(A) Correlation between expression of *VDAC1* and cytokines in PAAD datasets. **(B)** Correlation between expression of *VDAC1* and cytokines in LUAD datasets. *P* values were calculated by Spearman's rank correlation analysis, $P < 0.05$ was considered statistically significant

3. Supplemental Tables

Table S1. Datasets for identifing differentially expressed neural genes.

See Supplemental Tables file.

Table S2. Source of neural genes.

See Supplemental Tables file.

Table S3. Neural genes.

See Supplemental Tables file.

Table S4. Cancer-specific differentially expressed neural genes.

See Supplemental Tables file.

Table S5. Independent validation datasets.

See Supplemental Tables file.

Table S6 Spearman correlation between stage/grade and C-Neural scores.

See Supplemental Tables file.

Table S7. Pan-cancer scRNA-seq datasets.

See Supplemental Tables file.

Table S8. Datasets for melanoma and NSCLC by immunotherapy.

See Supplemental Tables file.

Table S9. The formulas of transcriptomic ICI signatures or algorithms from published literatures.

See Supplemental Tables file.

Table S10. Pharmacological screening data for cancer cell lines.

See Supplemental Tables file.

4. Abbreviations

Abbreviations	Definitions
AUC	area under the curve
BC	breast cancer
CAFs	cancer-associated fibroblasts
CCLE	Cancer Cell Line Encyclopedia
CIBERSORT	Cell-type Identification By Estimating Relative Subsets Of RNA Transcripts
C-Neural score	predictive score for Cancer-related Neural infiltration
CNV	copy number variation
COAD	colon adenocarcinoma
CopyKAT	Copy number Karyotyping of aneuploid Tumors
CPE	consensus measurement of purity estimations
CR	complete response
CR	partial response
CRC	colorectal cancer
CTRP	Cancer Therapeutics Response Portal
CytoTRACE	Cellular (Cyto) Trajectory Reconstruction Analysis using gene Counts and Expression
DEG-Ndown	differentially downregulated expressed neural genes
DEG-Ns	differentially expressed neural genes
DEG-Nup	differentially upregulated expressed neural genes
DepMap	Dependency Map portal
DFI	disease-free interval
DSS	disease-specific survival
EC50	half maximal effective concentration
ECM	extracellular matrix
EMT	epithelial-mesenchymal transition
epi-highCNs	epithelial cell with high C-Neural score
epi-lowCNs	epithelial cell with low C-Neural score
ESTIMATE	Estimation of STromal and Immune cells in MAlignant Tumours using Expression data
FC	fold change
FDA	The United States Food and Drug Administration
GBM	glioblastoma
GDSC	Genomics of Drug Sensitivity in Cancer
GEO	Gene Expression Omnibus
GO	Gene Ontology
GSA	Genome Sequence Archive
GSVA	gene set variation analysis
GTEx	The Genotype-Tissue Expression
H&E	hematoxylin & eosin
HLA	human leukocyte antigens
NSCLC	non-small cell lung cancer
HNSC	head and neck squamous cell carcinoma

IC50	half maximal inhibitory concentration
ICIs	immune checkpoint inhibitors
IHC	immunohistochemistry
iCAF	inflammatory CAF
KEGG	Kyoto Encyclopedia of Genes and Genomes
KIRC	kidney renal clear cell carcinoma
KIRP	kidney renal papillary cell carcinoma
LGG	low grade glioma
LUAD	lung adenocarcinoma
LUMP	leukocytes unmethylation for purity
LUSC	lung squamous cell carcinoma
MFS	metastasis-free survival
MIF	macrophage migration inhibitory factor
myCAF	myofibroblastic CAF
NE	not evaluated
NSCLC	non-small cell lung cancer
OS	overall surviva
OV	ovarian cancer
OXPHOS	oxidative phosphorylation
PAAD	pancreatic adenocarcinoma
PCA	principal component analysis
PCa	prostate cancer
PD	progressive disease
PDAC	pancreatic ductal adenocarcinoma
PFI	progression-free interval
PFS	progression-free survival
P-high$_t$	high C-Neural score population
P-low$_t$	low C-Neural score population
PNI	perineural invasion
ROC	receiver operating characteristic
PPI	protein-protein interaction
RECIST	Response Evaluation Criteria in Solid Tumors
RFS	relapse free survival
r-lCAF	reticular like CAF
RMA	Robust Multichip Average
scRNA-seq	single-cell RNA sequencing
SD	stable disease
SKCM	skin cutaneous melanoma
TCGA	The Cancer Genome Atlas
TMB	tumor mutational burden
TME	tumor microenvironment
TPM	transcripts per million
t-SNE	t-distributed stochastic neighbor embedding
tumor cell-highCNs	tumor cell with high C-Neural score
tumor cell-lowCNs	tumor cell with low C-Neural score

5. References

1. Hanahan D, Weinberg RA. The hallmarks of cancer, Cell 2000;100:57-70.
2. Hanahan D, Weinberg RA. Hallmarks of cancer: the next generation, Cell 2011;144:646-674.
3. Hanahan D. Hallmarks of Cancer: New Dimensions, Cancer Discov 2022;12:31-46.
4. Soltis AR, Bateman NW, Liu J et al. Proteogenomic analysis of lung adenocarcinoma reveals tumor heterogeneity, survival determinants, and therapeutically relevant pathways, Cell Rep Med 2022;3:100819.
5. Gillette MA, Satpathy S, Cao S et al. Proteogenomic Characterization Reveals Therapeutic Vulnerabilities in Lung Adenocarcinoma, Cell 2020;182:200-225 e235.
6. Kefeli J, Tatonetti N. TCGA-Reports: A machine-readable pathology report resource for benchmarking text-based AI models, Patterns (N Y) 2024;5:100933.
7. Petralia F, Wang L, Peng J et al. A new method for constructing tumor specific gene co-expression networks based on samples with tumor purity heterogeneity, Bioinformatics 2018;34:i528-i536.
8. Malta TM, Sokolov A, Gentles AJ et al. Machine Learning Identifies Stemness Features Associated with Oncogenic Dedifferentiation, Cell 2018;173:338-354 e315.
9. Newman AM, Liu CL, Green MR et al. Robust enumeration of cell subsets from tissue expression profiles, Nat Methods 2015;12:453-457.
10. Aran D, Sirota M, Butte AJ. Systematic pan-cancer analysis of tumour purity, Nat Commun 2015;6:8971.
11. Liu J, Lichtenberg T, Hoadley KA et al. An Integrated TCGA Pan-Cancer Clinical Data Resource to Drive High-Quality Survival Outcome Analytics, Cell 2018;173:400-416 e411.
12. Korsunsky I, Millard N, Fan J et al. Fast, sensitive and accurate integration of single-cell data with Harmony, Nat Methods 2019;16:1289-1296.
13. Xue M, Zhu Y, Jiang Y et al. Schwann cells regulate tumor cells and cancer-associated fibroblasts in the pancreatic ductal adenocarcinoma microenvironment, Nat Commun 2023;14:4600.
14. Cords L, Tietscher S, Anzeneder T et al. Cancer-associated fibroblast classification in single-cell and spatial proteomics data, Nat Commun 2023;14:4294.
15. Kim S, Leem G, Choi J et al. Integrative analysis of spatial and single-cell transcriptome data from human pancreatic cancer reveals an intermediate cancer cell population associated with poor prognosis, Genome Med 2024;16:20.
16. Hwang WL, Jagadeesh KA, Guo JA et al. Single-nucleus and spatial transcriptome profiling of pancreatic cancer identifies multicellular dynamics associated with neoadjuvant treatment, Nat Genet 2022;54:1178-1191.
17. Salcher S, Sturm G, Horvath L et al. High-resolution single-cell atlas reveals diversity and plasticity of tissue-resident neutrophils in non-small cell lung

cancer, Cancer Cell 2022;40:1503-1520 e1508.
18. Gao R, Bai S, Henderson YC et al. Delineating copy number and clonal substructure in human tumors from single-cell transcriptomes, Nat Biotechnol 2021;39:599-608.
19. Gulati GS, Sikandar SS, Wesche DJ et al. Single-cell transcriptional diversity is a hallmark of developmental potential, Science 2020;367:405-411.
20. Street K, Risso D, Fletcher RB et al. Slingshot: cell lineage and pseudotime inference for single-cell transcriptomics, BMC Genomics 2018;19:477.
21. Wu Y, Yang S, Ma J et al. Spatiotemporal Immune Landscape of Colorectal Cancer Liver Metastasis at Single-Cell Level, Cancer Discov 2022;12:134-153.
22. Aibar S, Gonzalez-Blas CB, Moerman T et al. SCENIC: single-cell regulatory network inference and clustering, Nat Methods 2017;14:1083-1086.
23. Jin S, Guerrero-Juarez CF, Zhang L et al. Inference and analysis of cell-cell communication using CellChat, Nat Commun 2021;12:1088.
24. Efremova M, Vento-Tormo M, Teichmann SA et al. CellPhoneDB: inferring cell-cell communication from combined expression of multi-subunit ligand-receptor complexes, Nat Protoc 2020;15:1484-1506.
25. Hu J, Zhang L, Xia H et al. Tumor microenvironment remodeling after neoadjuvant immunotherapy in non-small cell lung cancer revealed by single-cell RNA sequencing, Genome Med 2023;15:14.
26. Eisenhauer EA, Therasse P, Bogaerts J et al. New response evaluation criteria in solid tumours: revised RECIST guideline (version 1.1), Eur J Cancer 2009;45:228-247.
27. Hu FF, Liu CJ, Liu LL et al. Expression profile of immune checkpoint genes and their roles in predicting immunotherapy response, Brief Bioinform 2021;22.
28. Rydenfelt M, Wongchenko M, Klinger B et al. The cancer cell proteome and transcriptome predicts sensitivity to targeted and cytotoxic drugs, Life Sci Alliance 2019;2.
